# Parametrizing alternating current stimulation for neuromodulation

**DOI:** 10.1162/IMAG.a.1066

**Published:** 2026-01-07

**Authors:** Monica Bell Vila, Margaret Koletar, Adrienne Dorr, Andrea Trevisiol, Maged Goubran, John G. Sled, Paolo Bazzigaluppi, Bojana Stefanovic

**Affiliations:** Department of Medical Biophysics, University of Toronto, Toronto, ON, Canada; Physical Sciences, Sunnybrook Research Institute, Toronto, ON, Canada; Hurvitz Brain Sciences, Sunnybrook Research Institute, Toronto, ON, Canada; Harquail Centre for Neuromodulation, Sunnybrook Health Sciences Centre, Toronto, ON, Canada; Mouse Imaging Centre, Hospital for Sick Children, Toronto, ON, Canada; MetaCell S.r.L., Cagliari, Italy

**Keywords:** transcranial alternating current stimulation, neuromodulation, in situ electrophysiology

## Abstract

The clinical use of alternating current stimulation (ACS) has been confounded by heterogeneity in patient responses. Optimization of this treatment requires understanding the dependence of neuronal responses on ACS parameters. We sought to characterize neuronal responses across varying ACS frequencies and amplitudes and assess the extent to which changes in neuronal activity persist beyond the cessation of the stimulation, thereby informing ACS optimization pathways and evaluating the potential for sustained neuromodulation. We delivered subcutaneous ACS while recording intracortical electrophysiological activity in adult rats. We applied ACS for 1 or 20 minutes and analyzed ensuing spiking activity, power spectra, and low-frequency to high-frequency phase–amplitude coupling (LF-HF PAC). Both durations of ACS elicited a modulation of firing patterns (either in rate or entrainment) in most neurons, in a stimulation frequency- and amplitude-dependent manner. Changes in firing rate were more frequent, albeit modest, whereas changes in entrainment were rarer, but pronounced. Increasing ACS amplitude or frequency increased the degree of entrainment. Further, entrainment changes persisted beyond the 20-minute ACS, at half their online level. Neuronal power and LF-HF PAC increased proximal to excited, but decreased near suppressed neurons. Neuronal entrainment was observed alongside decreased power and LF-HF PAC. The prominent entrainment response to prolonged high-frequency ACS observed here supports the potential of ACS for facilitating signal integration and promoting functional reorganization via spike timing-dependent plasticity. In turn, we found broader temporal windows of excitation under low ACS frequencies engendered greater variability in spike timing, which may be utilized to reduce hypersynchronous or task-irrelevant activity.

## 1 Introduction

Transcranial alternating current stimulation (tACS) is a non-invasive method of delivering electric current to the brain that can synchronize spiking activity to a specific phase of the stimulation wave (Helfrich et al., 2014; W. A. Huang et al., 2021; Johnson et al., 2020; Krause et al., 2019). Such synchronization can have significant therapeutic potential given the significance of spike timing for neuronal plasticity (Caporale & Dan, 2008; Feldman, 2012; Markram et al., 2011). Unlike pharmacological interventions, ACS neuromodulation can be spatially confined and, through judicious selection of electrode montage and stimulation parameters, afford a non-invasive, low cost, safe, and portable way to functionally remodel the neuronal network and ameliorate brain dysfunction.

Notwithstanding, the clinical use of alternating current stimulation (ACS) has hitherto been confounded by high variability in brain responses to ACS observed in different studies (Héroux et al., 2017; Zanto et al., 2021). Although ACS has, in some studies, induced significant and lasting improvements in motor and memory tasks (Grover et al., 2022; Kasten & Herrmann, 2017; Lang et al., 2019; Miyaguchi et al., 2018; Nomura et al., 2019; Sale & Kuzovina, 2022; Sreeraj et al., 2019; Sugata et al., 2018), the elicited responses have varied with the frequency of ACS (Ali et al., 2013; Bologna et al., 2019; Guerra, Bologna, et al., 2018; Janssens et al., 2022; Klink et al., 2020; Nakazono et al., 2020; Wöstmann et al., 2018; Zhang et al., 2022), but mechanisms underlying this frequency selectivity remain unclear, confounding systematic optimization. While many studies demonstrate persistent effects of ACS, it is unclear whether these offline effects are caused by persistence of the imposed patterns of entrainment (i.e., increases in rhythmic activity of neurons at the ACS frequency delivered), which may require more frequent or sustained delivery to maintain, or by the modulation of synapses via the induction of spike timing plasticity (Johnson et al., 2020; Strüber et al., 2015; Vossen et al., 2015), resulting in network reorganization and self-subsisting effects. Finally, whether ACS can modulate cortical excitability—in addition to eliciting entrainment—remains unclear: some studies reported post-ACS changes in neuronal power spectrum and firing rates in addition to entrainment (Reato et al., 2010; Sugata et al., 2018; Tran et al., 2022; Vossen et al., 2015; Wischnewski et al., 2019; Zaehle et al., 2010), while others observed changes only in spike timing (Johnson et al., 2020; Krause et al., 2019). Given the broad set of neuronal dysfunction patterns seen in brain diseases, understanding which aspects of the pathology-induced aberration can be normalized by ACS treatment would be particularly valuable for maximizing its therapeutic efficacy (van Bueren et al., 2021).

Tailoring stimulation parameters requires understanding the dependence of the neuronal response on ACS parameters. Different amplitudes of ACS likely recruit different neural mechanisms: whereby low magnitude electric fields bias the probability of a spike occurring over a temporal window, stronger fields impose new spiking patterns, synchronizing the endogenous rhythmic activity to that of the stimulus, affecting both the timing and rate of firing (Abd Hamid et al., 2015; A. Liu et al., 2018). Stimulation frequency is a key parameter as exceeding neurons’ peak firing rates may limit neuronal engagement due to insufficient repolarization between induced depolarizations (Lowet et al., 2022). *In vitro* (Lee et al., 2024) and modeling work (X. Huang et al., 2024) suggest that a frequency selectivity of ACS arises from class-specific cellular properties, thus potentially enabling targeting of different neuronal subtypes, which are often differentially affected in brain diseases (Dauer & Przedborski, 2003; Frahm et al., 2004; Y.-Q. Liu et al., 2014; Loup et al., 2000; MacDonald & Halliday, 2002; Motaharinia et al., 2021; Palop & Mucke, 2016; Pasquereau & Turner, 2011; Saiz-Sanchez et al., 2015; Underwood & Parr-Brownlie, 2021; Verret et al., 2012; Wang, 2003; Xu et al., 2020; Zhan et al., 2006).

To provide a broad interpretation on the variability of neuronal response across ACS parameters, including the proportion of neurons affected within the field of stimulation as well as the degree of their modulation, we delivered 1-minute trials of frequency- and amplitude-varying subcutaneous ACS (sACS) to anesthetized adult rats and analyzed the ensuing somatosensory neuronal activity measured via an intracortical multi-electrode planar array. Stimulation frequencies were selected to probe endogenous oscillatory bands (θ: 5 Hz, α: 10 Hz, β: 20 Hz, and γ: 40 Hz) and four amplitudes (50, 100, 200, 400 μA), with the lowest amplitude selected for its ability to induce electric fields of 1 mV/mm, reported as the minimal magnitude needed to alter mammalian brain’s spiking patterns (Kang et al., 2020; Vöröslakos et al., 2018). This electric field is clinically achievable with currents of 4–6 mA (Y. Huang et al., 2017; Vöröslakos et al., 2018; Wischnewski et al., 2023), which, though higher than the currently accepted safety threshold of 2 mA, has been administered with mild to no adverse effects (Chhatbar et al., 2017; Khadka et al., 2020; Workman et al., 2019), and can also be attained with multi-electrode placement (Alekseichuk et al., 2019). To assess whether observed neuromodulatory effects persisted beyond stimulation offset, we delivered 20 minutes of sACS at 40 Hz (which, of frequencies tested in the 1-minute paradigm, yielded strongest entrainment) and analyzed electrophysiological signals in the 10 minutes succeeding sACS offset. We thereby mapped the frequency and amplitude dependence in the neuronal response to sACS, and quantified the neuronal activity changes that endure past stimulation cessation, providing the first *in situ* characterization of the dependence of the cortical response across ACS parameters (amplitude, frequency, and duration).

## 2 Methods

### 2.1 Animals

In total, 22 adult Sprague-Dawley rats were used in this study. Nine 3-month-old Sprague-Dawley rats (4 male, 5 female) were used for 1-minute sACS trials for parameter exploration, and 13 rats (7 male, 6 female) for 20-minute sACS experiments to assess offline effects. All rats were housed in standard cages on ventilated racks, with food and water available *ad libitum*, on a 12-hour light–dark cycle. Experimental procedures followed the ARRIVE (Animal Research: Reporting of In Vivo Experiments) guidelines (Percie du Sert et al., 2020) and were approved by and performed in accordance with the regulations established by the Animal Care Committee of the Sunnybrook Research Institute, which adheres to the Policies and Guidelines of the Canadian Council on Animal Care (CCAC) and meets all the requirements of the Provincial Statute of Ontario, Animals for Research Act as well as those of the Federal Health of Animals Act, and the International Council for Laboratory Animal Science (ICLAS).

### 2.2 Animal preparation

Rats were anesthetized with isofluorane (5% induction, 2–2.5% maintenance) and secured in a stereotaxic apparatus (SR-50, Narishige International, USA). Physiological monitoring was carried out using a pulse oximeter (MouseOx Plus, Starr Life Sciences Corp, USA) to monitor heart rate, respiration rate, and arterial oxygen saturation, via a hind paw probe. An electric heating pad with a thermorectal feedback probe (Temperature Controller TC-1000, CWE Inc, USA) maintained core body temperature at 37°C. An intravenous tail vein catheter was placed for delivery of propofol anesthesia during electrophysiological recordings as functional connectivity under propofol sedation has been demonstrated to better reflect that of awake rats relative to that of rats under isofluorane (Paasonen et al., 2018). Skin was excised to expose the dorsal skull surface. A craniotomy was performed using a high-speed drill (Foredom Electric Co., Bethel, Connecticut, USA) to create an opening between bregma and lambda over the right hemisphere (AP -1.0 to -7.0 mm, ML +1.0 to +5.5 mm). The dura was resected to expose the pial surface. The right lateral temporal muscle was removed to allow placement of the stimulating electrode. Following surgical preparation, isofluorane was discontinued. A bolus of propofol (7.5 mg/kg) was administered, followed by continuous infusion (44.0 mg/kg/hr) via the tail vein catheter. Propofol infusion rate was adjusted so that rats were maintained in a lightly sedated state, established through the monitoring of heart and respiratory rates. To minimize electromagnetic interference in recordings, the stereotaxic apparatus was enclosed within a Faraday cage.

### 2.3 Electrophysiology

The 16-platinum iridium recording shanks had a tip width of 125 μm with an impedance of 0.4 MOhm, and were spaced 0.5 mm apart in the lateral plane. A final shank with 10 kOhm impedance was used as a reference. Data were sampled at 24 kHz (PZ5 Neurodigitizer Amplifier, Tucker-Davis Technologies), with local field potentials (0.3–300 Hz, with notch filters at 60, 120, and 180 Hz) stored at 12 kHz, and multi-unit activity (500–3000 Hz) stored at 24 kHz.

### 2.4 sACS parameters

A craniotomy exposed the right sensorimotor cortex (AP -1.0 to -7.0 mm, ML +1.0 to +5.5 mm), and a 4 × 4 multielectrode array (Microprobes) was lowered 300 μm into layer 3 (Markram et al., 2015) for electrophysiological recordings. The two copper and polyamide electrode arrays (Neuronexus), with 1.5 × 1.5 mm gold-plated stimulating pads, were adhered subcutaneously, directly on the skull, on either side of the cranial window (AP -4.0 mm, ML 0.0 mm and AP -4.0 mm, ML +5.2 mm, DV -3.0 mm) using conductive adhesive hydrogel (Tensive Parker Labs). The electrodes were further secured using a 3D printed holder glued to the skull (with Bondic).

**Fig. 1.**
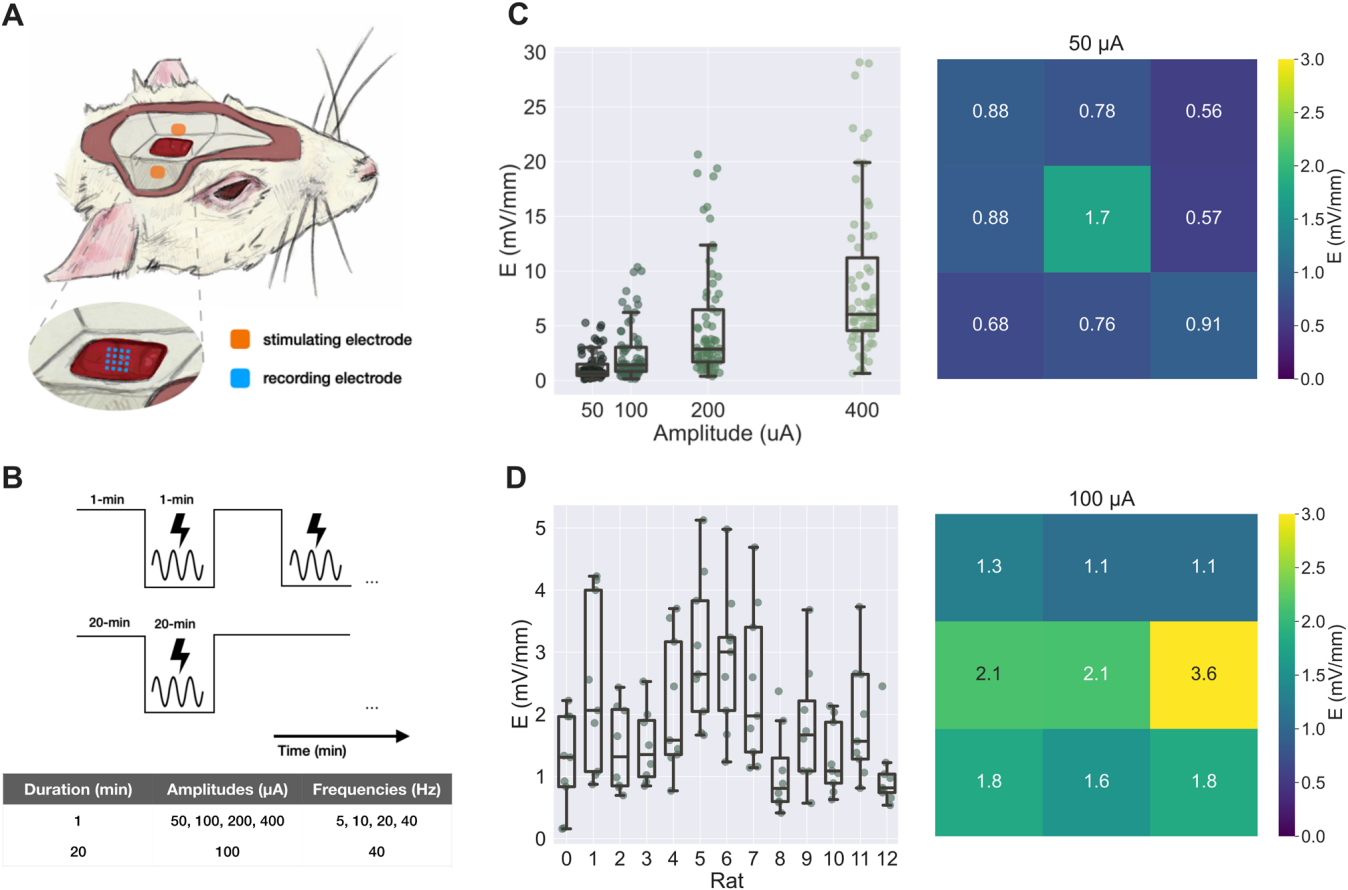
Experimental Setup. Custom copper and polyamide electrode (Neuronexus) comprising 1.5 × 1.5 mm gold-plated stimulating pads, with 1.0 mm spacing, were secured medial and lateral to the cranial window (A) in propofol-anesthetized rats with a 3D-printed electrode holder. A 4 × 4 multielectrode array (Microprobes) was lowered 300 μm below the cortical surface, into layer 3 of the exposed sensorimotor cortex. For 1-minute sACS experiments, all combinations of stimulation frequencies (5, 10, 20, and 40 Hz) and amplitudes (50, 100, 200, and 400 μA) were delivered in a randomized order, with 1 minute baseline recording before each stimulation trial (B). For 20-minute sACS experiments, 100 μA, 40 Hz stimulation was delivered for 20 minutes following 20 minutes of baseline recording. (C) The electric fields computed between channels for all rats across 1-minute sACS paradigms (left). A heatmap showing the median electric fields across the horizontal plane across rats for 50 μA sACS (right). (D) Distributions of electric field estimates for all rats from the 100 μA 20-minute sACS experiments (left). A heatmap showing the median electric fields across the horizontal plane from these experiments (right).

For parameter exploration, propofol-anesthetized rats underwent 20 stimulation trials, each consisting of 1 minute of baseline, 1 minute of stimulation, and 1 minute of post-stimulus baseline, probing a range of ACS frequencies (5, 10, 20, 40, 140 Hz) and amplitudes (50, 100, 200, 400 μA, or 22.2, 44.4, 88.8, and 177.6 A/m^2^), delivered in a randomized order (Fig. 1). A stimulation period of 1 minute was selected for sACS parameter exploration experiments given its demonstrated ability to induce online effects (Mencarelli et al., 2022), while being sufficiently short to enable return to baseline/recovery (Johnson et al., 2020), thus enabling the investigation of a broad range of parameter combinations in the same animal. Stimulation frequencies (5, 10, 20, 40, 140 Hz) were selected so as to probe endogenous oscillatory bands (θ: 4–8 Hz, α: 8–12 Hz, β: 12–30 Hz, low-γ: 30–80 Hz, high-γ: >80 Hz), and the lowest stimulation current amplitude (50 μA) was chosen to produce the electric field magnitude of 1 mV/mm, commonly accepted as the minimum level required to induce changes in mammalian brain’s spiking patterns (Kang et al., 2020; Vöröslakos et al., 2018).

To assess whether neuromodulatory effects persisted beyond sACS offset, rats underwent a single trial of 40 Hz, 100 μA sACS applied for 20 minutes. This frequency was selected for its effectiveness in inducing entrainment in the 1-minute stimulation paradigm, at an amplitude of 100 μA, which is well tolerated for the 20-minute ACS in awake rats (Yu et al., 2015), yet substantially more effective than 50 μA (Baba et al., 2009; Vöröslakos et al., 2018; Wachter et al., 2011). We again looked at online changes in spiking patterns, but also assessed them in the 10 minutes following stimulation offset. To infer modulations in network activity, we computed the power spectra as well as the modulation index (MI) (Combrisson et al., 2020; Tort et al., 2010), a measure of low-frequency to high-frequency phase–amplitude coupling (LF-HF PAC) from the local field potential (LFP) of channels proximal to sACS-affected neurons. Linear mixed effects modeling was used to examine contrasts in the log-transformed power and order normalized (Peterson & Cavanaugh, 2020) MI between pre- and post-stimulus conditions.

For a sham stimulation condition, recordings were acquired from a single F344 nTg female rat which underwent the same surgical preparation as in stimulation conditions, but was maintained under propofol anesthetic without the delivery of sACS for 80 minutes.

### 2.5 Electrophysiological data analyses

ACS produces a sinusoidal artifact in electrophysiological recordings at the applied stimulation frequency and its harmonics, compromising the fidelity of local field potential (LFP: 0.3–300 Hz) recordings during the stimulation period. As spiking activity occurs at frequencies much higher than that of the main component of the ACS artifact, the multi-unit activity band (MUA: 500–3000 Hz) activity was assessed by first attenuating the ACS artifact during stimulation periods using a weighted causal uniform comb filter (ARtACS software; Guggenberger, n.d.). Notwithstanding, artifact removal was incomplete in 140 Hz sACS frequency trials, so these trials were dropped from subsequent analysis. Consequently, MUA analysis was conducted for the entire duration of the 1- and 20-minute trials, whereas LFP analysis was performed only on recordings pre- and post-stimulation of 20-minute trials. LFP signals were decimated to 1.2 kHz.

Spike times of neurons were extracted from the multi-unit band activity (MUA: 500–3000 Hz; Fig. 3) using SpyKING CIRCUS (Yger et al., 2018), and were subsequently analyzed to measure sACS-induced changes from baseline in firing rate (FR) and entrainment (Fig. 4), estimated using the square root of the absolute pairwise phase consistency (PPC) (Vinck et al., 2010). Significantly affected neurons were detected using permutation tests, with linear regressions performed across sACS amplitudes and frequencies to test for parameter dependence.

### 2.6 Firing rate (FR) and pairwise phase consistency (PPC)

Automated spatial whitening (Cohen, 2022; Yger et al., 2018) and spike sorting were performed on per trial MUA recordings with SpyKING CIRCUS software (Yger et al., 2018), using the software recommended median absolute deviation threshold of 6 for spike detection. Detected spikes were clustered by merging templates having greater than 0.95 correlation. For each detected neuron, we computed the number of spikes over 10-second windows across the baseline and stimulation periods.

Neurons exhibiting sparse FRs (<0.25 Hz) in more than half of the windows were discarded to ensure a sufficient number of spikes (>10 over 1-minute windows) were available for accurate estimates of the pairwise phase consistency in the subsequent analysis (Vinck et al., 2010). For each stimulation trial, significant changes were assessed comparing the 1-minute stimulation period against the immediately preceding 1-minute of baseline.

FRs of neurons were computed based on the number of detected spikes over 1-minute observation periods, averaging across non-overlapping 1-second windows. To determine the phase locking value, the Hilbert transform was first applied to the stimulating waveform to estimate the instantaneous phase of the signal over time. Spike times for pre-stimulus baseline and during stimulus periods of each neuron, as reported by SpyKING CIRCUS, were independently compared against this array to extract corresponding phases. Since the conventional computation of the phase locking value (PLV), abs (mean(exp(iθ))) where θ rresents the recorded phases, depends on the number of detected spikes and is thus positively biased (Mc Laughlin et al., 2022), the square root of the absolute pairwise phase consistency (PPC), an unbiased estimate of the PLV, was computed for all neurons as the cosine of the absolute angular distances, that is, the average vector dot product, across all pairs of detected phases (Vinck et al., 2010). Thus, the equation for PLV estimates, the √|PPC|, used was $\sqrt{\;\left|\frac{2}{N(N-1)}\sum_{j=1}^{N-1}\sum_{k=(j+1)}^{N}cos(\theta_{j})cos(\theta_{k})+sin(\theta_{j})sin(\theta_{k})\right|}$. A PLV or √|PPC| value of 0 denotes no entrainment, while a value of 1 indicates complete entrainment. An example of entrainment is displayed in Figure 4.

A permutation test was used to test which neurons showed statistically significant changes in and √|PPC| metrics (Jean-Richard-Dit-Bressel et al., 2020; Johnson et al., 2020; Maris & Oostenveld, 2007). The FR was computed over 100-ms bins over the pre-stimulus baseline and during stimulation periods for each neuron. The FR bins were then pooled together, shuffled, and resampled to generate surrogate pre- and during stimulation groups, from which the average FRs from each group were used to determine a pseudo ΔFR consistent with the null hypothesis. This process was iterated 10,000 times to create a simulated null distribution, against which the true ΔFR between pre- and during stimulation periods was compared. A neuron was deemed responsive if its absolute ΔFR was observed in ≤5% of null distributions (p ≤ 0.05). Similarly, a significant change in √|PPC| was determined by pooling and shuffling phases at which spikes occurred pre- and during stimulation periods, resampling into surrogate pre- and during stimulation groups. The √|PPC| was computed from the phases of the two groups, and the difference between the surrogate pre- and during stimulation groups used to determine the pseudo Δ√|PPC|. This process was repeated 10,000 times to create an estimate of the null distribution, against which the true Δ√|PPC| was compared, and again deemed significantly responsive if occurring in ≤5% of null distribution samples (p ≤ 0.05). A Grubb’s test was applied to Δ√|PPC| and ΔFR values for each frequency/amplitude combination of sACS parameters to remove outliers from each group (α = 0.05).

To test for sACS parameter dependence on the number of affected neurons (i.e., those showing changes in their firing rates or entrainment), linear mixed effects modeling (LMER) was conducted in R (version 4.3.3). For the subset of neurons with significantly altered spike patterns (√|PPC| or FR) under sACS, the change from the preceding 1 minute of baseline (Δ√|PPC| or ΔFR) was modeled as the dependent variable, with the amplitude and frequency of stimulation included as interacting independent variables. To account for across-subject and across-neuron variability, the subject and neuron were modeled as random effects, nesting the neuron within the subject. In addition, linear regressions were performed on the altered spike patterns of affected neurons across all sACS amplitudes and frequencies, computed using the scipy.stats software package in Python, and a threshold probability of ≤5% under a null hypothesis was once again used to ascertain significance (p ≤ 0.05).

For the 20-minute ACS experiments, significant changes in neuronal activity were similarly detected following the aforementioned procedures. Significance testing was performed comparing the full 20 minutes of stimulation against the full 20 minutes of baseline as well as comparing the 10 minutes immediately following stimulation against the 10 minutes immediately preceding the stimulation.

### 2.7 Power spectra and low-frequency to high-frequency phase–amplitude coupling (LF-HF PAC)

For the 20-minute ACS experiments, the LFP of electrode channels proximal to neurons with significantly increased or decreased ΔFR or Δ√|PPC| during the full duration of the 20-minute stimulation period was selected for analysis. Channels associated with mixed responses (increases and decreases in ΔFR/Δ√|PPC|) were excluded to better isolate trends associated with local inhibition versus excitation/entrainment versus decreased entrainment.

A non-overlapping fast Fourier transform was applied to 2-second recording windows over 2-minute periods immediately pre- and post-stimulus to estimate neuronal power. For each window, power levels within each frequency band (δ: 0.5–4, θ: 4–8, α: 8–12, β: 12–30, low-γ: 30–80, high-γ: 80–140 Hz) were averaged and, given the 1/frequency^2^ scaling observed in intracortical LFP power (Baranauskas et al., 2012), log-transformed. To visualize changes in power spectra over time, spectrograms were computed for each of the 2-minute periods immediately pre- and post-stimulus. For each of the four conditions considered (increased/decreased ΔFR or Δ√|PPC|), spectrograms were averaged across all channels proximal to associatively behaving neurons.

The modulation index (MI), a measure of dependence of the high frequency (30–140 Hz) band’s amplitude on the low frequency (1–29 Hz) band’s phase, was computed (in 1 Hz steps with 5 Hz width windows for high frequencies, and 0.5 Hz steps with 2 Hz widths for low frequencies) following the method described by Tort et al. (2010) using the Tensorpac software (Combrisson et al., 2020). Similarly to power spectra, the MI was computed over the 2-minute periods immediately pre- and post-stimulus, but in 10-second windows, the minimum window length affording robust estimation (Dvorak & Fenton, 2014). To perform MI estimations, a continuous wavelet transform was first applied to extract the phase time series, φ(*t*), as well as the amplitude envelope, A(*t*). The phase values were divided into 18 bins and the average amplitude over each phase bin was computed. The MI was then calculated from these amplitudes as the Kullback–Leibler divergence from a uniform distribution of amplitude across phases (Combrisson et al., 2020; Tort et al., 2010). The amplitude time series was repeatedly shuffled and analyzed by the same method described previously to obtain 200 surrogate amplitude envelopes, creating a distribution of the amplitude time series in the absence of LF-HF PAC. MI values of the original amplitude time series were deemed significant if they resided in the top 5% of the distribution of MI values computed from the shuffled surrogate distributions without LF-HF PAC. For each window, mean MI values were obtained for all low-frequency (δ: 0.5–4, θ: 4–8, α: 8–12, β: 12–29) to high-frequency (low-γ: 30–80, high-γ: 80–140 Hz) couplings.

Linear mixed effects modeling (LMER, using lme4; Bates et al., 2015) was conducted in R (version 4.3.3) to examine the contrast in our electrophysiological metrics between pre- and post-stimulus conditions. For channels with affected FR or PPC, the mean log-power or the order normalized mean MI values were modeled as the dependent variable, with the frequency band (or cross band relationship for MI values), condition (pre- or post-stimulus), and associated channel response (increase or decrease in entrainment/FR) included as independent variables. To account for across-subject and across-electrode channel variability, subject and channel were modeled as random effects, nesting the channel within the subject. Pairwise comparisons were performed on the estimated marginal means (emmeans; Lenth, 2024) between pre- and post-stimulus groups for all combinations of frequency bands and channel response (increase or decrease in entrainment or FR), with a Bonferroni correction applied across pre- and post-comparisons.

The sham sACS recording was subdivided into sixteen 3-minute blocks, and each block assigned to a randomized combination of the 1-minute stimulation parameters (amplitudes of 50, 100, 200, and 400 μA and frequencies of 5, 10, 20, and 40 Hz). Each 3-minute block was analyzed to compare 1-minute of baseline against the 1-minute of sham stimulation following the same analysis pipeline as in the 1-minute sACS pipeline, whereby significantly affected neurons during the sham stimulation period were “detected.” Further, the power spectra and modulation indices across frequency bands of the LFP recording of the sham experiment were computed using the same 3- (power) and 10-s (modulation index) windows as used in 20-min sACS analysis paradigms. We looked at the average values and standard deviations in 1-minute windows every 10 minutes across the sham experiment.

#### 2.8 Electric field estimations

To acquire experimentally derived electric field estimates for each sACS amplitude (50, 100, 200, and 400 μA), electrophysiological activity for all channels of each subject from all stimulation trials of that amplitude was bandpass filtered to isolate the stimulation frequency (5, 10, 20, and 40 Hz for 1-minute paradigms and 40 Hz for 20-minute paradigms). Over the stimulation period, we averaged the peak recorded voltage magnitude over each stimulation cycle at the respective electrode channel’s location. Given the 4 × 4 layout of the multielectrode array, we calculated the difference in the aforementioned average peak voltages between neighboring electrodes along both axes and divided this difference by the distance between electrodes to compute the maximal electric fields in both directions (Y. Huang et al., 2017; Opitz et al., 2015, 2018). The orthogonal electric field estimates were then combined using vector addition. A Grubb’s test was applied to electric field values to remove outliers (α = 0.05). The average orthogonal electric field was computed across all frequency stimulation periods for each amplitude for each rat.

FEM was conducted using Sim4Life software, adapting the available ViZOO Big Male Rat model (Kainz et al., 2006). A Boolean mask was used to subtract experimentally resected areas of skin, muscle, and connective tissue around the skull. Stimulation electrodes were modeled as 1.5 × 1.5 × 0.1 mm gold volume boxes projecting onto the surface of the skull. Electric fields were computed using the Electro Ohmic Quasi-Static Solver with a maximum step size of 0.5 × 0.5 × 0.5 mm, increased to 0.1 × 0.1 × 0.1 mm between stimulating electrodes, and geometry resolution of 10 × 10 × 10 mm. The current passed between stimulation electrodes was scaled to the experimentally tested amplitudes (50, 100, 200, and 400 μA), and overall electric fields across space were exported for subsequent analysis. In Python, the PyVista software (Sullivan & Kaszynski, 2019) was used to scale the root mean square (RMS) estimates of the electric field such that values in the simulation matched the experimentally measured values in the 2 × 2 mm grid space of the 4 × 4 MEA approximately 300 μm below the cortical surface. Log-normal distributions were fitted to cerebral electric field RMS values using the scipy.stats package. RMS values were thresholded at 1 mV/mm, and ellipsoids were fitted to the resulting volumes, using the *pyvista.principal_axes* function to extract the standard deviation of surface points along each dimension. These standard deviation values were then multiplied by a factor 1.96 (so as to encompass 95% of the surface) as ellipsoid axes radii.

## 3 Results

### 3.1 Firing rate modulations were common but limited in magnitude, while entrainment levels were less frequent but pronounced

**Fig. 2.**
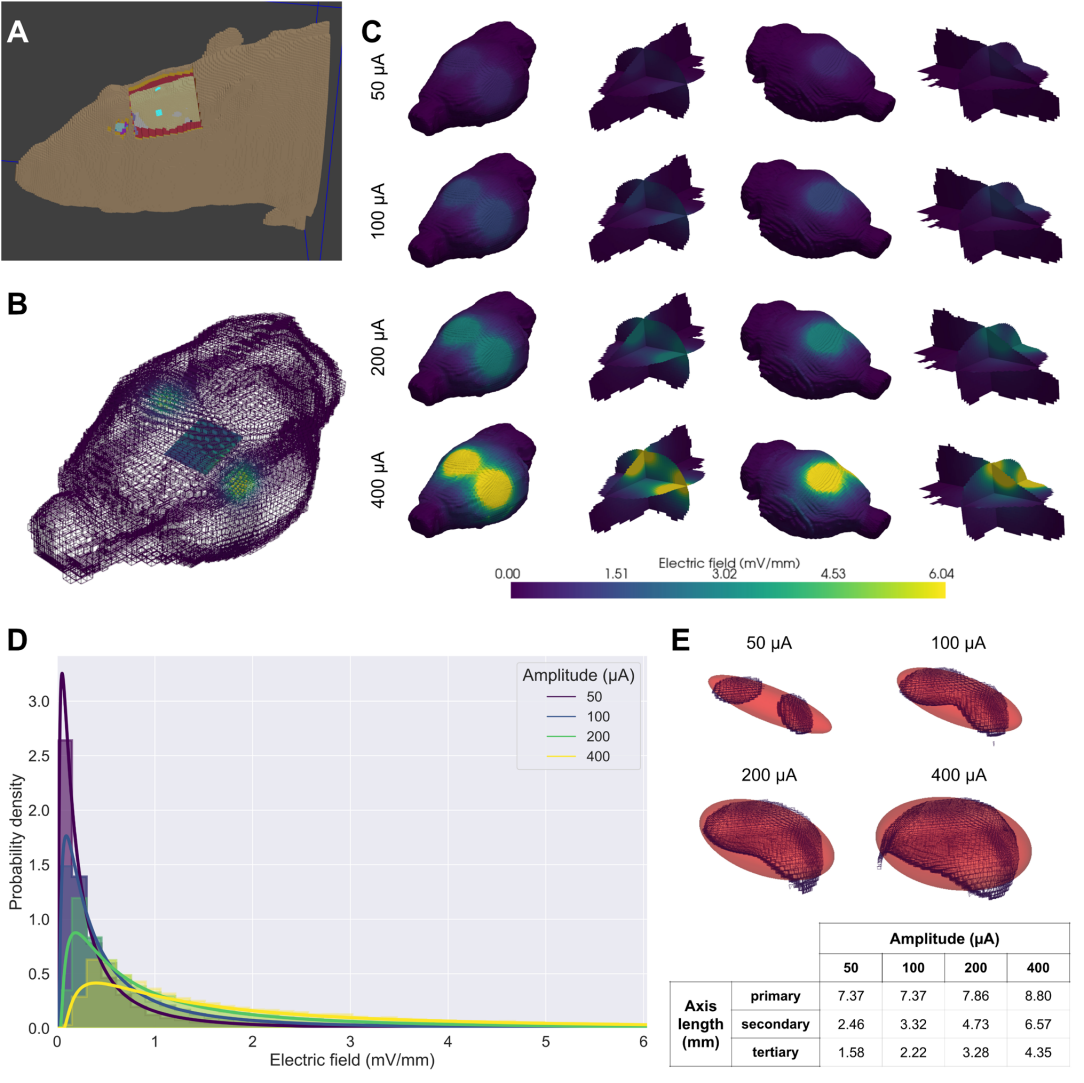
FEM electric field estimations. (A) Voxels of rat head used for FEM in Sim4Life software. A Boolean subtraction was used to remove experimentally resected areas of skin, muscle, and connective tissue around the skull. Stimulation electrodes with dimensions matching experimental conditions (1.5 × 1.5 mm) are displayed as blue squares. (B) RMS estimates of the electric field generated via FEM were normalized such that values in the simulation matched the experimentally measured values in the 2 × 2 mm grid space of the 4 × 4 MEA approximately 300 µm below the cortical surface. (C) Whole brain and orthogonal slice views of the estimated electric field distributions across the brain for each of the four amplitudes tested experimentally. The electric field drops to ~50% of the intensity measured at the recording electrodes at a further ~70 µm in depth. (D) Histograms showing electric field probability densities within the cerebral hemispheres for stimulation amplitudes. Log-normal distributions were fitted to cerebral electric field RMS values and plotted, with shape, location, and scale parameters of 1.28, 8.54E-03, 2.17E-01 for 50 µA, 1.28, 1.57E-02, 4.00E-01 for 100 µA, 1.28, 3.17E-02, 8.07E-01 for 200 µA, and 1.28, 6.70E-02, 1.70 for 400 µA. (E) The cerebral hemispheres were thresholded to identify regions containing an electric field magnitude of at least 1 mV/mm. A 3D ellipsoid was fit to these volumes, with primary, secondary, and tertiary axes lengths of 7.37, 2.46, and 1.58 mm for 50-µA ACS; 7.37, 3.32, and 2.22 for 100-µA ACS; 7.86, 4.73, and 3.28 mm for 200-µA ACS; and 8.80, 6.57, and 4.35 mm for 400-µA ACS.

For parameter exploration, propofol-anesthetized rats underwent a series of stimulation trials, each consisting of 1 minute of baseline, 1 minute of stimulation, and 1 minute of post-stimulus baseline, with parameters delivered in a randomized order (Fig. 1A, B). Stimulation frequencies (5, 10, 20, and 40 Hz) were selected so as to probe endogenous oscillatory bands (θ: 4–8, α: 8–12, β: 12–30, low-γ: 30–80 Hz), and increasing stimulation amplitudes were investigated (50, 100, 200, 400 μA, or 22.2, 44.4, 88.8, and 177.6 A/m^2^). The magnitude of electric fields was assessed from sACS artifacts in electrophysiological recordings, and had medians of 0.77 ± 0.29, 1.42 ± 0.60, 2.86 ± 1.20, and 6.04 ± 1.68 mV/mm for 50, 100, 200, 400 μA sACS amplitudes, respectively (Fig. 1C). Finite element modeling (FEM) using Sim4Life software was used to estimate and visualize electric fields produced across the brain across stimulation amplitudes (Fig. 2). Within the model, the electric field dropped to ~50% of the intensity measured at the recording electrodes at a further ~70 µm in depth. The cerebral electric field root mean square (RMS) values were fitted with log-normal distributions, with shape, location, and scale parameters of 1.28, 8.54E-03, 2.17E-01 for 50 µA; 1.28, 1.57E-02, 4.00E-01 for 100 µA; 1.28, 3.17E-02, 8.07E-01 for 200 µA; and 1.28, 6.70E-02, 1.70 for 400 µA. Ellipsoids were fit to thresholded hemispheric volumes experiencing electric field magnitude of at least 1 mV/mm, with primary, secondary, and tertiary axes lengths of 7.37, 2.46, and 1.58 mm for 50-µA ACS; 7.37, 3.32, and 2.22 for 100-µA ACS; 7.86, 4.73, and 3.28 mm for 200-µA ACS; and 8.80, 6.57, and 4.35 mm for 400-µA ACS.

**Fig. 3.**
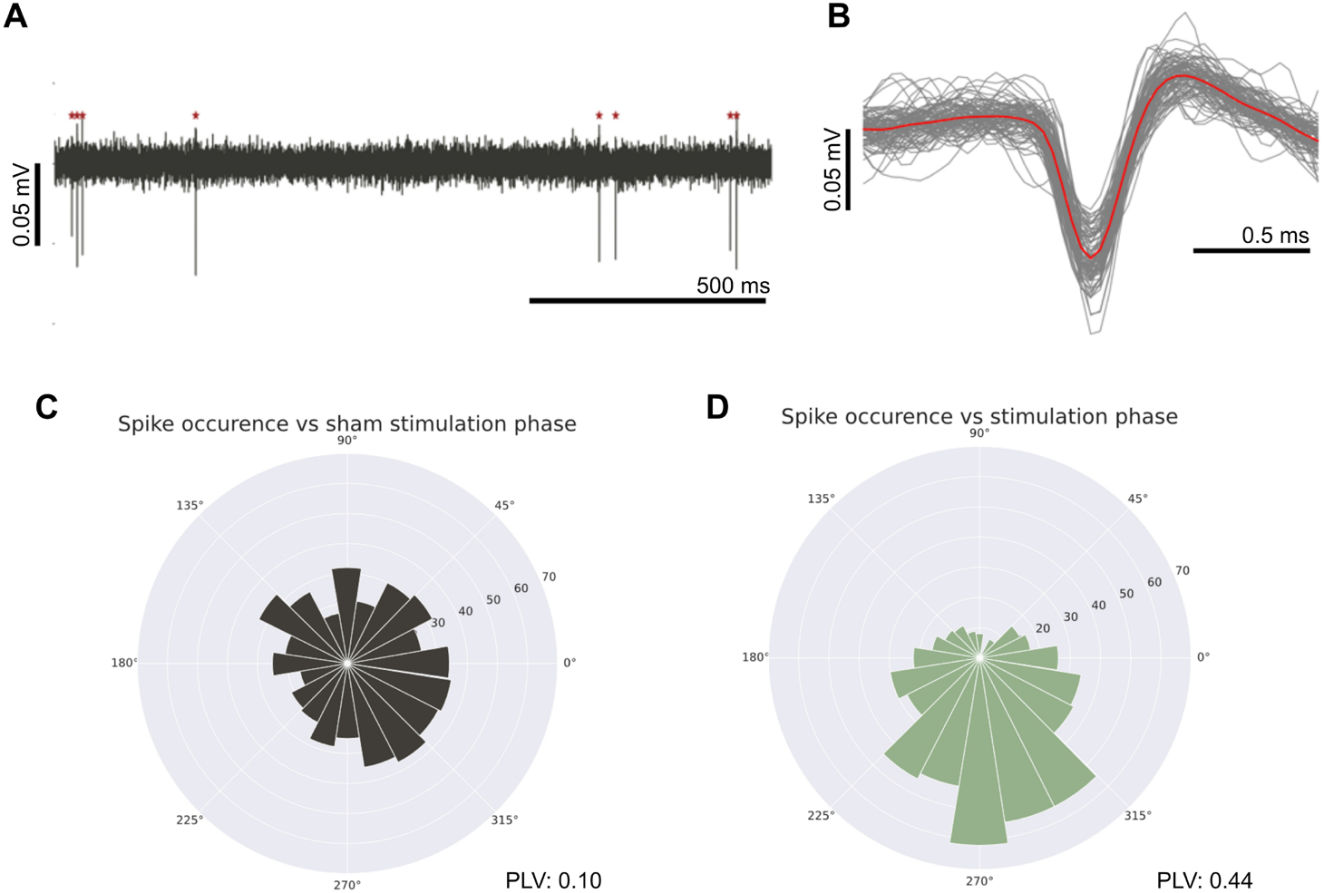
Representative multi-unit activity (MUA) traces and relative phase distribution at baseline versus upon sACS-induced entrainment. (A) MUA (500–3000 Hz) recording from a single-electrode channel. Downward inflections detected as spiking activity by Spyking Circus (≥6 MAD threshold) are denoted with red stars. (B) Spiking activity assigned to a neuron: all spike waveforms are shown in gray with median activity trace in red. Spike timing relative to the phase of stimulating waveform for a single neuron before (C) and during (D) sACS. (C) At baseline, spikes occur at any phase relative to the “temporally extended” sACS waveform. (D) During sACS, spikes are more concentrated at a specific phase of the stimulating waveform (here 270 degrees).

**Fig. 4.**
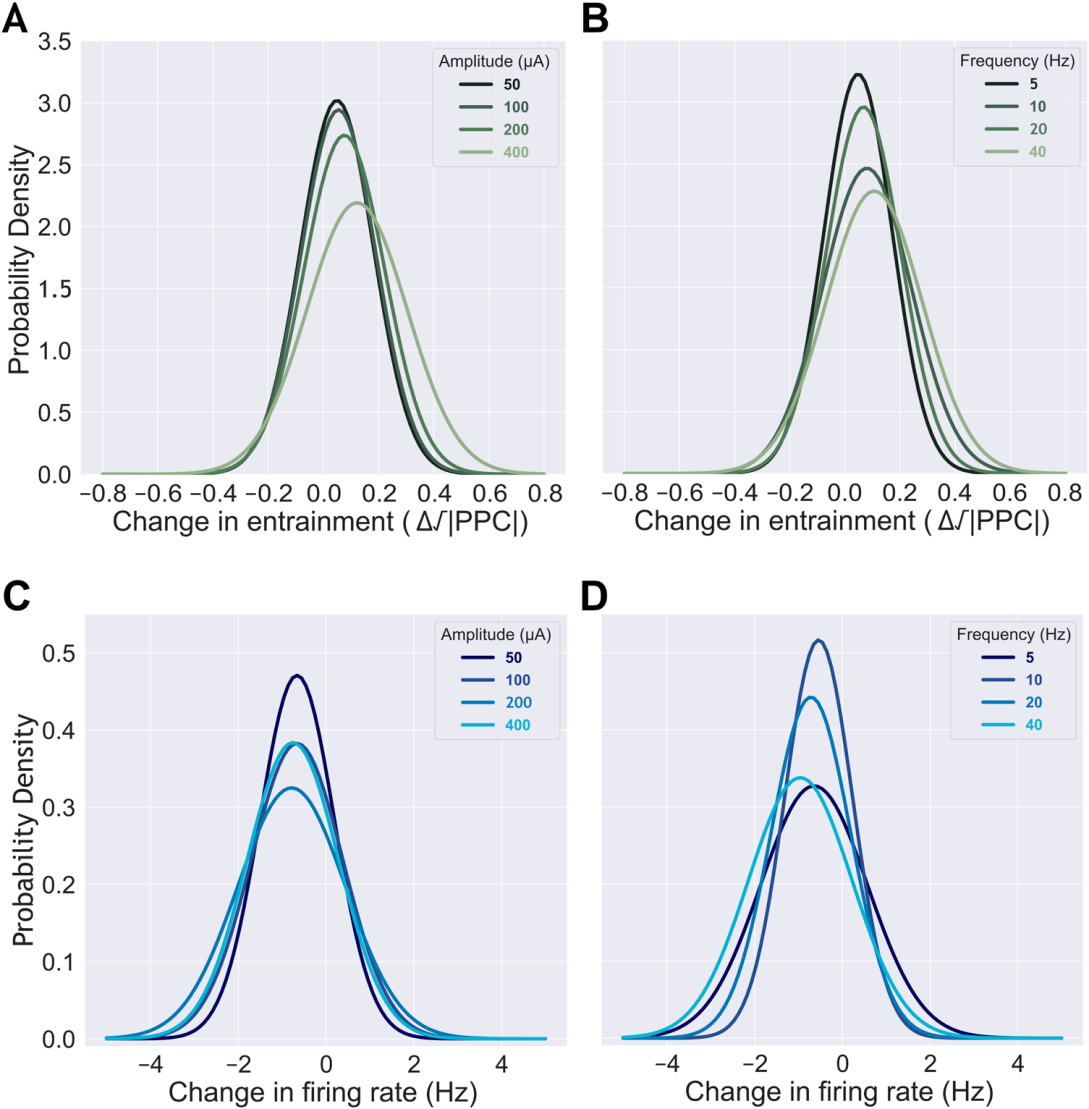
The dependence of neurons’ responses on sACS parameters. The dependence of the magnitude of entrainment and firing rate on sACS amplitude (A, C) and frequency (B, D) when contrasting 1-minute sACS to 1 minute of baseline. Probability density functions fit to the distribution of changes in entrainment (A, B) and firing rate (C, D) of all neurons from their baseline under sACS. With increasing sACS amplitude (A) and frequency (B), neurons exhibit a broader distribution of responses, with a shift toward stronger entrainment from baseline and a slight suppression of their activity (C, D).

**Fig. 5.**
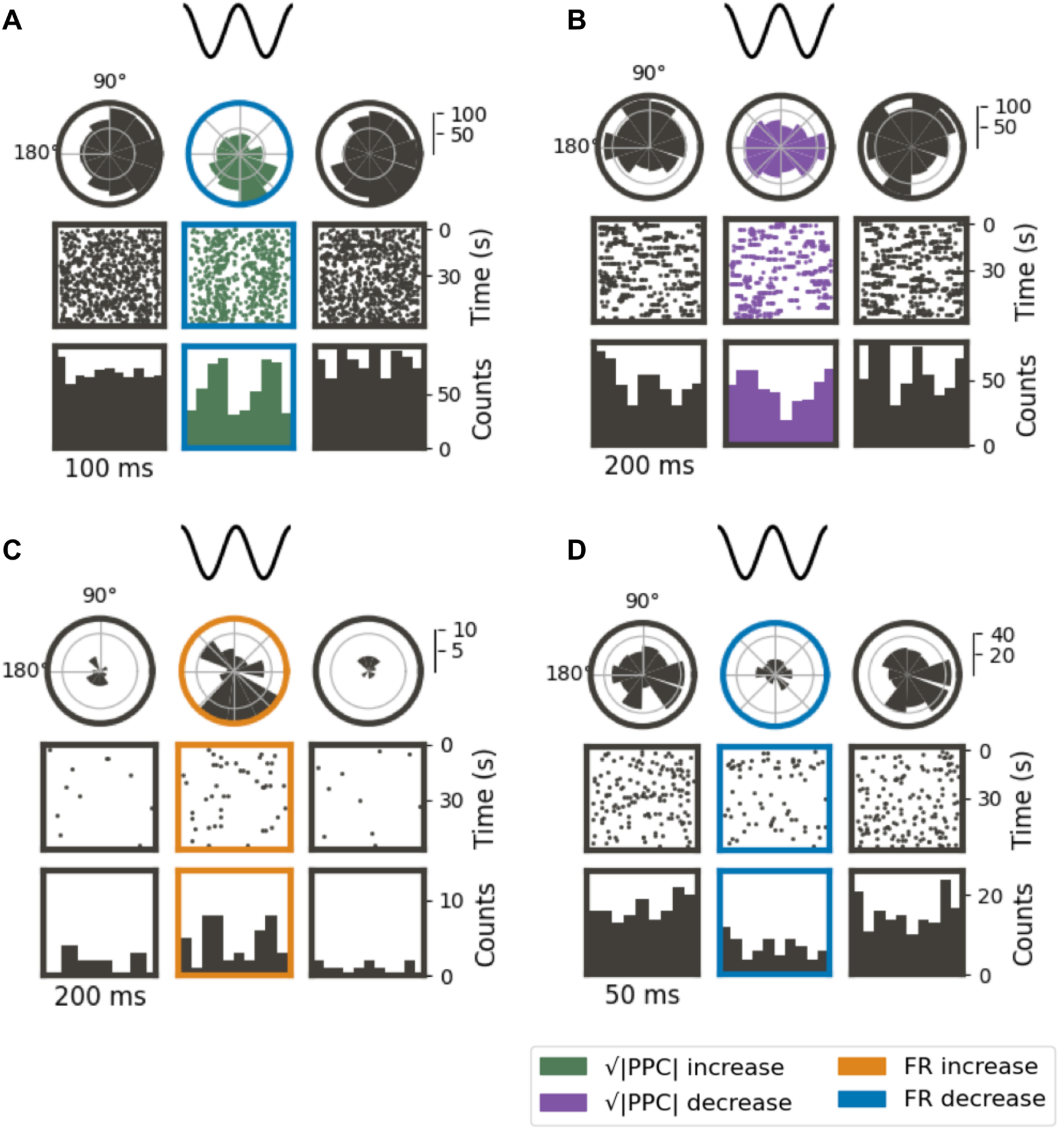
Representative neuronal responses during 1 minute of sACS. Each subplot represents a single neuron, with the three columns showing behavior over 1-minute periods of pre-stimulus baseline, sACS stimulation period, and the period immediately succeeding sACS offset. The first row contains spike density histograms versus phase; the second row, spike rasters; and the final row, spike density histograms versus time. Examples are shown of neurons exhibiting increased entrainment (A); decreased entrainment (B); increased FR (C); decreased FR (D).

**Fig. 6.**
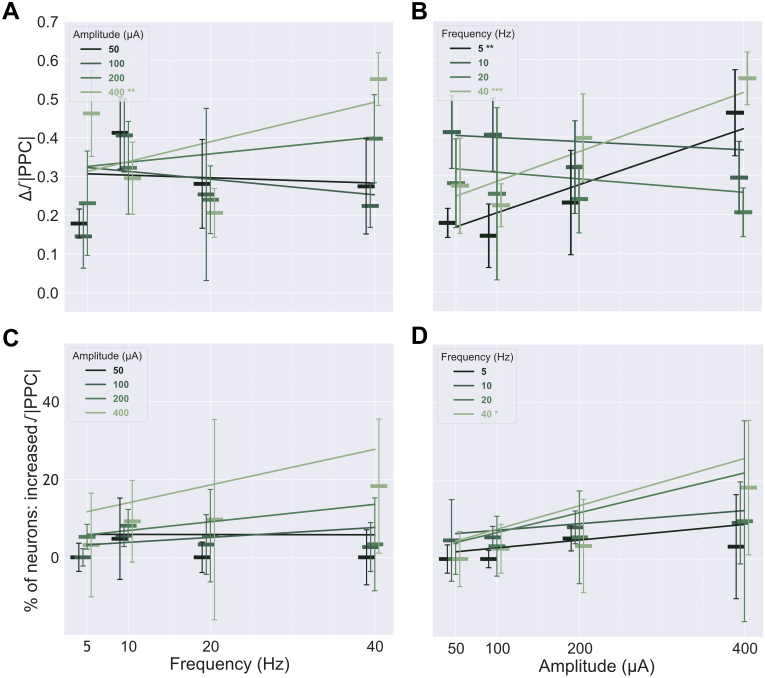
The dependence of the positively entrained neurons’ responses on sACS parameters. The dependence of the magnitude of entrainment, that is, the change in Δ√|PPC|, on sACS frequency (A) and amplitude (B) when contrasting 1-minute sACS to 1 minute of baseline. The dependence of the percentage of neurons found to be significantly entrained (p = 0.05) of all detected neurons firing at ≥0.25 Hz during sACS trials across frequency (C) and amplitude (D). Bars show median values, with the 95% CI indicated with error bars. The 400 µA linear regressions showed significant increases in Δ√|PPC| with increasing frequency at 5.10 × 10^−3^ Δ√|PPC| per Hz, p = 0.002) (A). Δ√|PPC| increased with increasing amplitude for 5- and 40-Hz stimulations (7.22 × 10^−4^ and 7.63 × 10^−4^ Δ√|PPC| per μA, p = 0.004 and 0.0002, respectively) (B), and 40-Hz stimulation frequency demonstrated an increase in the number of neurons entrained with increasing amplitude (6.04 × 10^−2^ % per μA, p = 0.007) (D).

**Fig. 7.**
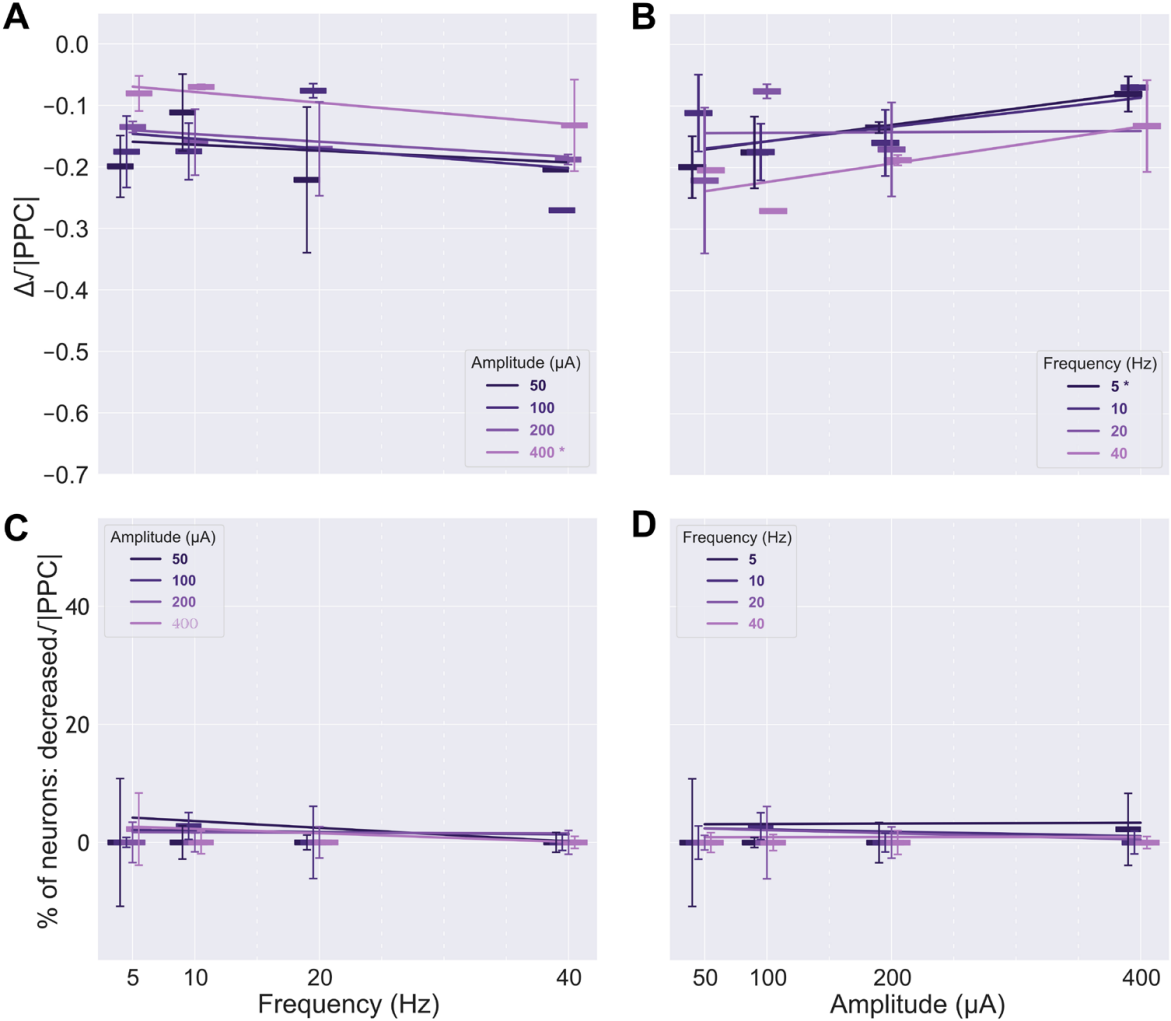
The dependence of the negatively entrained neurons’ responses on sACS parameters. The dependence of the magnitude of entrainment, that is, the change in Δ√|PPC|, on sACS frequency (A) and amplitude (B) when contrasting 1-minute sACS to 1 minute of baseline. The dependence of the fraction of neurons found to have significantly decreased entrainment (p = 0.05) of all detected neurons firing at ≥0.25 Hz during sACS trials across frequency (C) and amplitude (D). Bars show median values, with the 95% CI indicated with error bars. In (A) and (C), linear regressions are shown for different stimulation amplitude conditions against sACS frequency. In (B) and (D), linear regressions are shown for different stimulation frequency conditions against sACS amplitude. Only the 400-µA regression (A) across frequencies and the 5-Hz regression across amplitudes (B) showed significance, with -1.74 × 10^−3^ Δ√|PPC| per Hz and 2.67 × 10^−4^ Δ√|PPC| per µA (p = 0.04 and 0.005, respectively).

**Fig. 8.**
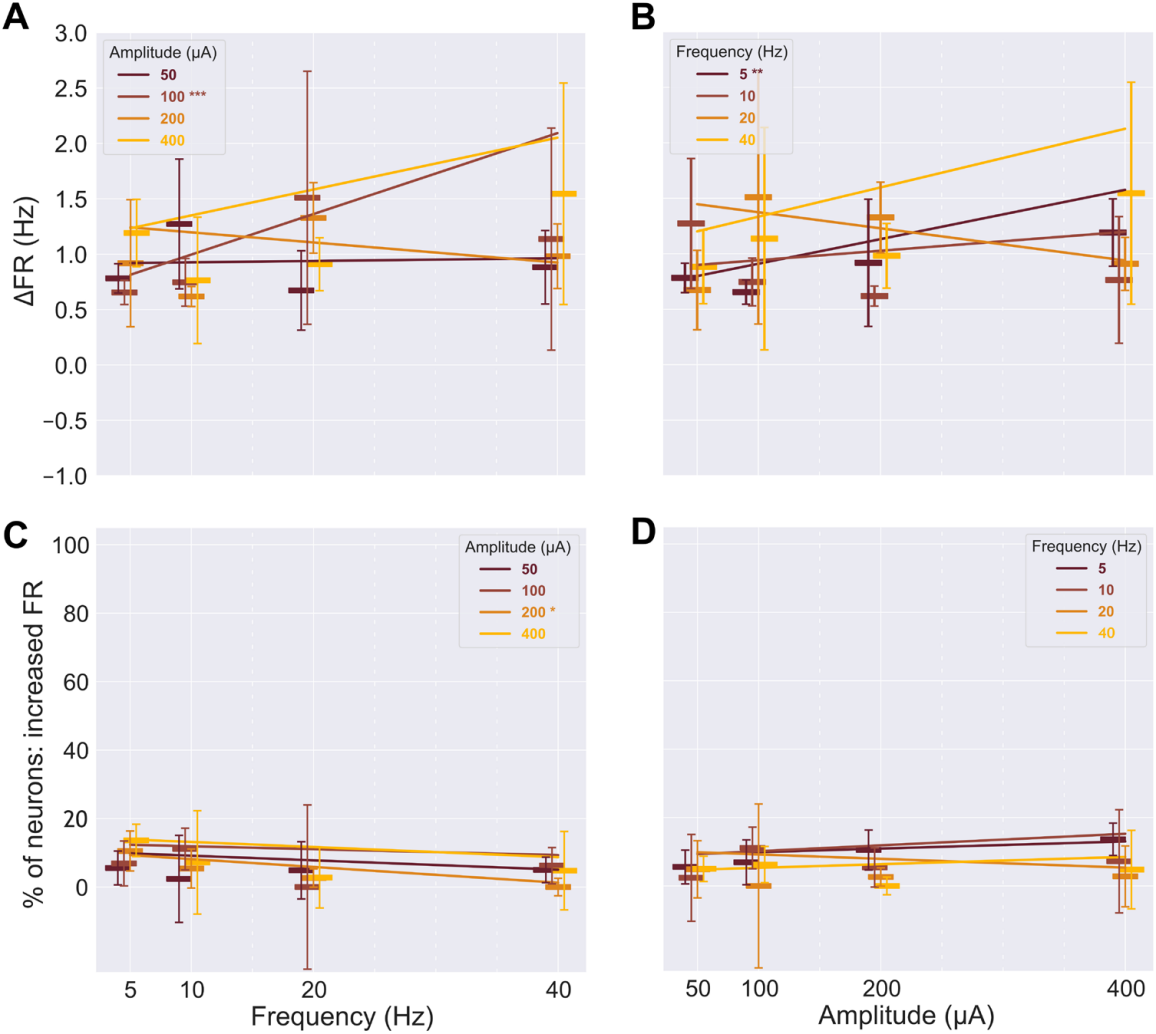
Responses across stimulation parameters for neurons that showed a significant increase in firing rate during sACS. The dependence of FR, that is, the change in ΔFR, on sACS frequency (A) and amplitude (B) when contrasting 1-minute sACS to 1 minute of baseline for excited neurons. The dependence of the percentage of neurons found to have significantly increased FRs (p = 0.05) of all detected neurons firing at ≥0.25 Hz during sACS trials across frequency (C) and sACS amplitude (D). Bars show median values, with the 95% CI indicated with error bars. In (A) and (C), linear regressions are shown for different stimulation amplitude conditions against sACS frequency. In (B) and (D), linear regressions are shown for different stimulation frequency conditions against sACS amplitude. The 100-µA regression across frequencies (A) showed a significant increase in the ΔFR of excited neurons with increasing frequency (3.65 × 10^−2^ ΔHz per Hz, p = 0.0003), whereas the 5-Hz regression across amplitudes (B) showed an increase in the ΔFR of excited neurons with increasing amplitude (2.22 × 10^−3^ ΔHz per μA, p = 0.002). The 200-µA regression across frequencies considering the percentage of excited neurons (C) showed a significant decrease with increasing frequency (-2.30 × 10^−3^ % per Hz, p = 0.01).

**Fig. 9.**
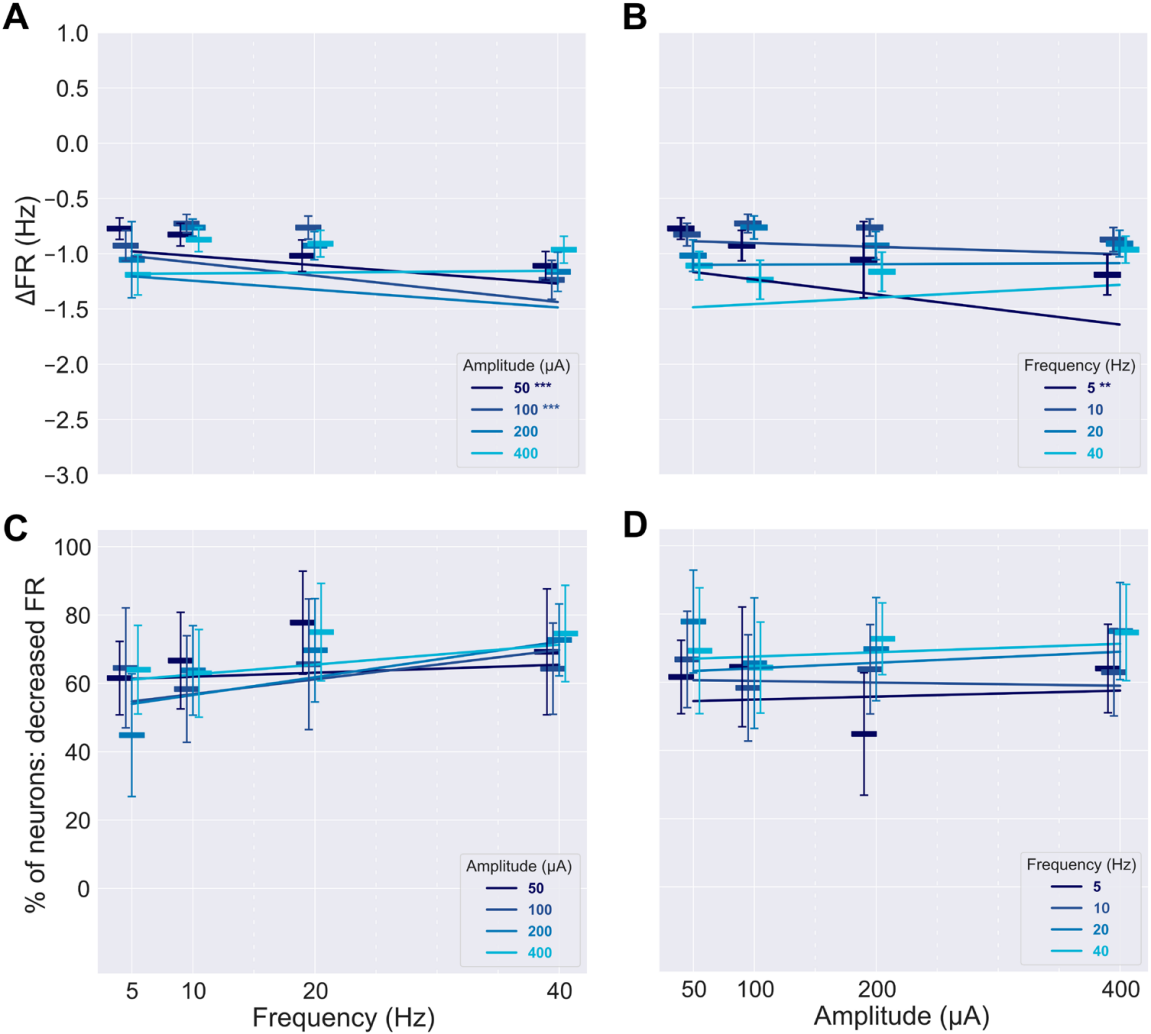
Responses across stimulation parameters for neurons that showed a significant decrease in firing rate during sACS. The dependence of FR, that is, the change in ΔFR, on sACS frequency (A) and amplitude (B) when contrasting 1-minute sACS to 1 minute of baseline for suppressed neurons. The dependence of the percentage of neurons found to have significantly decreased FRs (p = 0.05) of all detected neurons firing at ≥0.25 Hz during sACS trials across frequency (C) and sACS amplitude (D). Bars show median values, with the 95% CI indicated with error bars. In (A) and (C), linear regressions are shown for different stimulation amplitude conditions against sACS frequency. In (B) and (D), linear regressions are shown for different stimulation frequency conditions against sACS amplitude. The 50 and 100 µA regressions across frequencies (A) showed greater degrees of suppression in FRs with increasing frequency (-8.33 × 10^−3^ and -1.19 × 10^−2^ ΔHz per Hz, p = 0.0004 and <0.0001, respectively). Only the 5-Hz regression (B) showed increased suppression with increasing amplitude (-1.36 × 10^−3^ ΔHz per µA, p = 0.0008).

**Fig. 10.**
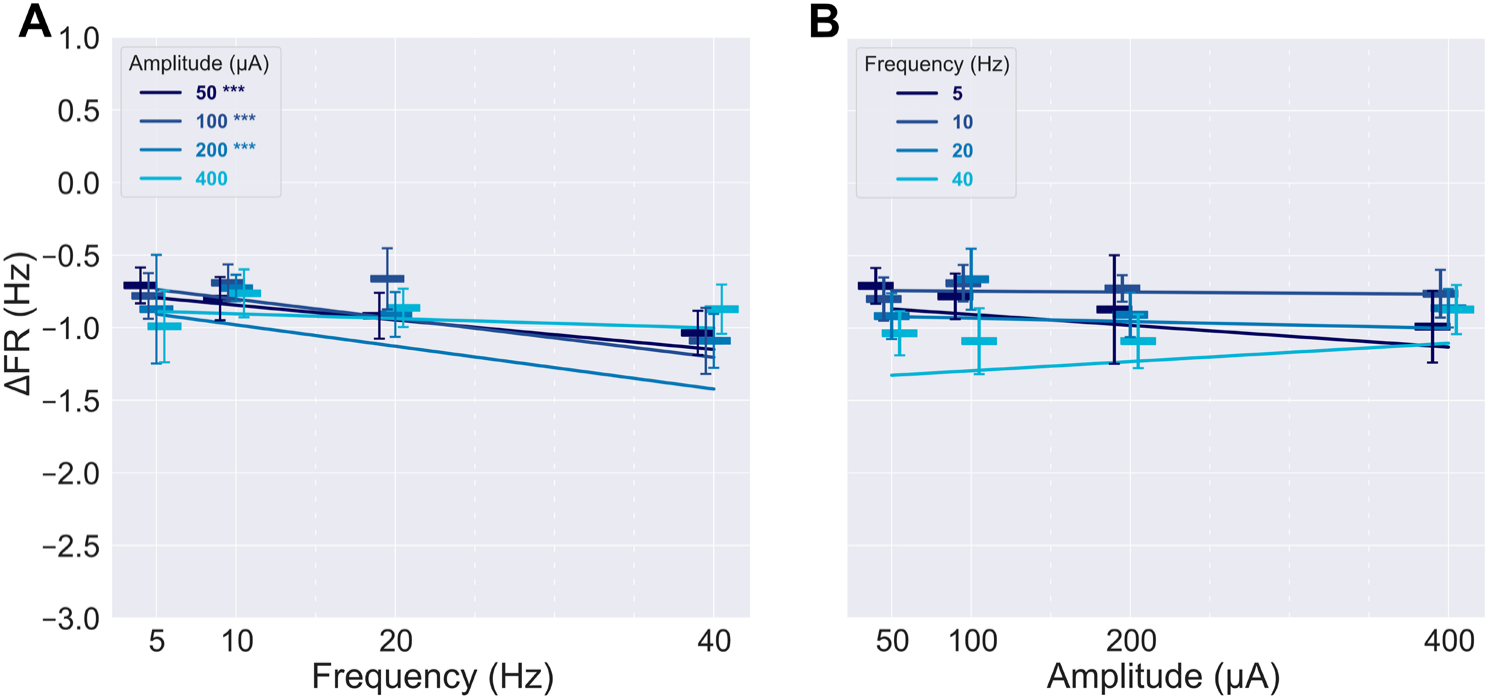
Responses across stimulation parameters for neurons that showed any significant change in firing rate during sACS. The dependence of FR, that is, the change in ΔFR, on sACS frequency (A) and amplitude (B) when contrasting 1-minute sACS to 1 minute of baseline for both excited and suppressed neurons. Bars show median values, with the 95% CI indicated with error bars. In (A) linear regressions are shown for different stimulation amplitude conditions against sACS frequency. In (B) linear regressions are shown for different stimulation frequency conditions against sACS amplitude. The 50, 100, and 200 µA regressions across frequencies (A) showed enhanced suppression of FRs with increasing frequency (-1.02 × 10^−2^, -1.33 × 10^−2^, and -1.47 × 10^−2^ ΔHz per Hz, p = 0.0003, <0.0001, and 0.0001, respectively).

Across rats, a median of 5.00 ± 0.17 neurons were detected per recording electrode channel using SpyKING CIRCUS (Yger et al., 2018) software (Fig. 3A, B). This amounted to 66.00 ± 19.60 neurons per rat, of which 71.43 ± 10.39% (or, cumulatively, 405/586) had FRs ≥0.25 Hz in at least half of 10-second windows (of baseline and stimulation periods) and were, therefore, included in subsequent analysis. Significant changes in neuronal entrainment and FRs (Figs. 4 and 5) were induced in some neurons by 1-minute sACS irrespectively of its frequency and amplitude, but the degree of firing pattern change as well as the number of neurons affected varied across parameters (Tables 1–3; Figs. 6–10; Supplementary Tables S1.1–S1.5; Supplementary Figs. S1.3–S1.6). Across trials, about 75% of neurons were affected (amplitude/frequency-specific percentages are listed in Table 1). A change in FR was more frequent than a change in spike timing (approximately 70% vs. <10%; Table 1); FR remained within ~2 Hz of baseline level (Table 2), 1.20 ± 2.11 Hz (Supplementary Fig. S1.1), whereas entrainment levels achieved were pronounced (Δ√|PPC| of up to 0.55; c.f., Table 2), changing from baseline levels of 0.12 ± 0.05, 0.12 ± 0.07, 0.11 ± 0.10, 0.11 ± 0.09 to 5, 10, 20, and 40 Hz endogenous oscillatory frequencies, respectively (Supplementary Fig. S1.2). The FR change was predominantly negative (net inhibition, Figs. 4C, D and 10; Supplementary Fig. S1.5); whereas the vast majority of spike timing affected neurons showed increases in entrainment (4%, Table 1; Figs. 4A, B and 6; Supplementary Fig. S1.3); and only a few neurons exhibited negative Δ√|PPC| values (<0.5%; Table 1; Fig. 7; Supplementary Fig. S1.4): that is, a decrease in the synchronization to endogenous rhythmic activity at the frequency matching that of the applied stimulus (Krause et al., 2022; Zhao et al., 2023). LFP analysis did not show significant changes in power or LF-HF PAC before and after 1-min sACS (Supplementary Tables S1.6–S1.7), suggesting this duration of stimulation is not sufficient to produce offline network effects.

**Table 1.** Percentages of neurons affected across 1-minute stimulation paradigms.

			Amplitude (µA)				Frequency medians (across all amplitudes)
			50	100	200	400	
Frequency (Hz)	5	*Any change (%)* *√|PPC| increase (%)* *√|PPC| decrease (%)* *FR increase (%)* *FR decrease (%)*	74.07 ± 11.95 0.00 ± 3.62 0.00 ± 10.82 5.56 ± 4.94 61.54 ± 10.76	71.43 ± 12.70 0.00 ± 2.23 0.00 ± 0.85 6.90 ± 6.56 64.52 ± 17.55	57.89 ± 19.47 5.26 ± 3.19 0.00 ± 3.41 10.53 ± 5.84 44.83 ± 17.96	75.93 ± 13.69 3.17 ± 13.31 2.24 ± 6.11 13.62 ± 4.73 64.00 ± 12.97	69.23 ± 7.45 1.85 ± 1.76 0.00 ± 3.41 8.78 ± 4.93 59.26 ± 7.68
	10	*Any change (%)* *√|PPC| increase (%)* *√|PPC| decrease (%)* *FR increase (%)* *FR decrease (%)*	80.00 ± 8.90 4.76 ± 10.45 0.00 ± 2.82 2.38 ± 12.71 66.67 ± 14.13	73.68 ± 11.47 5.56 ± 2.80 2.78 ± 2.27 11.11 ± 6.10 58.33 ± 15.59	74.14 ± 11.31 8.11 ± 4.19 0.00 ± 1.59 5.41 ± 5.70 63.79 ± 13.09	77.10 ± 7.12 9.26 ± 10.49 0.00 ± 1.92 7.19 ± 15.10 62.92 ± 12.81	76.89 ± 6.91 6.00 ± 2.47 1.39 ± 1.33 6.48 ± 9.31 65.38 ± 6.77
	20	*Any change (%)* *√|PPC| increase (%)* *√|PPC| decrease (%)* *FR increase (%)* *FR decrease (%)*	84.21 ± 9.21 0.00 ± 3.91 0.00 ± 1.24 4.88 ± 8.35 77.78 ± 15.06	71.96 ± 11.98 3.28 ± 7.66 0.00 ± 6.12 0.00 ± 23.98 65.59 ± 19.13	75.76 ± 10.84 5.56 ± 11.87 0.00 ± 2.65 2.63 ± 2.66 69.70 ± 15.12	81.82 ± 7.96 9.68 ± 25.70 0 2.78 ± 8.90 75.00 ± 14.24	80.00 ± 6.76 4.00 ± 8.63 0.00 ± 0.29 1.32 ± 7.73 69.70 ± 7.79
	40	*Any change (%)* *√|PPC| increase (%)* *√|PPC| decrease (%)* *FR increase (%)* *FR decrease (%)*	73.08 ± 13.13 0.00 ± 7.06 0.00 ± 1.68 5.00 ± 3.71 69.23 ± 18.44	85.00 ± 10.77 2.63 ± 6.27 0.00 ± 1.36 6.25 ± 5.28 64.29 ± 13.35	77.42 ± 5.87 3.33 ± 11.93 0.00 ± 2.02 0.00 ± 2.56 72.73 ± 10.51	84.03 ± 8.85 18.28 ± 17.22 0.00 ± 1.02 4.79 ± 11.48 74.58 ± 14.10	77.94 ± 7.09 5.46 ± 8.36 0.00 ± 0.88 4.98 ± 2.87 70.00 ± 6.96
	Amplitude medians (across all frequencies)	*Any change (%)* *√|PPC| increase (%)* *√|PPC| decrease (%)* *FR increase (%)* *FR decrease (%)*	74.01 ± 9.68 3.07 ± 2.38 0.72 ± 1.41 4.94 ± 6.74 69.70 ± 13.77	72.81 ± 7.69 2.17 ± 2.65 0.38 ± 0.75 5.26 ± 5.77 65.59 ± 17.20	72.88 ± 7.54 4.70 ± 5.0 0.62 ± 1.21 4.78 ± 3.21 69.85 ± 11.82	84.93 ± 7.35 11.11 ± 13.21 0.43 ± 0.85 7.41 ± 8.34 70.97 ± 10.70	74.19 ± 8.42 4.17 ± 2.61 0.00 ± 0.44 6.70 ± 5.38 69.23 ± 12.64

For each amplitude and frequency combination of sACS, the median and 95% confidence interval of the percentage of neurons per rat that were detected as being significantly affected from the neurons with firing at rates of ≥0.25 Hz for that trial.

**Table 2.** Changes in firing patterns for neurons affected across 1-minute stimulation paradigms.

			Amplitude (µA)				Frequency medians (across all amplitudes)
			50	100	200	400	
Frequency (Hz)	5	*√|PPC| increase* *√|PPC| decrease* *FR increase (Hz)* *FR decrease (Hz)*	0.18 ± 0.04 -0.20 ± 0.05 0.78 ± 0.13 -0.77 ± 0.10	0.15 ± 0.08 -0.18 ± 0.06 0.65 ± 0.11 -0.93 ± 0.14	0.23 ± 0.13 -0.14 ± 0.01 0.92 ± 0.57 -1.05 ± 0.35	0.46 ± 0.11 -0.08 ± 0.03 1.19 ± 0.30 -1.19 ± 0.18	0.18 ± 0.06 -0.13 ± 0.03 0.85 ± 0.17 -0.95 ± 0.10
	10	*√|PPC| increase* *√|PPC| decrease* *FR increase (Hz)* *FR decrease (Hz)*	0.41 ± 0.09 -0.11 ± 0.06 1.27 ± 0.59 -0.83 ± 0.10	0.41 ± 0.09 -0.17 ± 0.05 0.75 ± 0.22 -0.73 ± 0.08	0.32 ± 0.12 -0.16 ± 0.05 0.62 ± 0.09 -0.76 ± 0.08	0.30 ± 0.09 -0.07 ± 0.00 0.76 ± 0.57 -0.87 ± 0.11	0.36 ± 0.05 -0.13 ± 0.03 0.65 ± 0.23 -0.80 ± 0.05
	20	*√|PPC| increase* *√|PPC| decrease* *FR increase (Hz)* *FR decrease (Hz)*	0.28 ± 0.11 -0.22 ± 0.12 0.67 ± 0.36 -1.02 ± 0.14	0.25 ± 0.22 -0.08 ± 0.01 1.51 ± 1.14 -0.76 ± 0.10	0.24 ± 0.09 -0.17 ± 0.08 1.33 ± 0.32 -0.93 ± 0.13	0.21 ± 0.06 n/a 0.91 ± 0.24 -0.91 ± 0.12	0.25 ± 0.05 -0.13 ± 0.05 0.98 ± 0.40 -0.91 ± 0.06
	40	*√|PPC| increase* *√|PPC| decrease* *FR increase (Hz)* *FR decrease (Hz)*	0.27 ± 0.12 -0.20 0.88 ± 0.33 -1.11 ± 0.13	0.22 ± 0.06 -0.27 1.14 ± 1.00 -1.24 ± 0.18	0.40 ± 0.11 -0.19 ± 0.01 0.98 ± 0.29 -1.16 ± 0.18	0.55 ± 0.07 -0.13 ± 0.07 1.55 ± 1.00 -0.96 ± 0.12	0.39 ± 0.05 -0.19 ± 0.05 1.09 ± 0.45 -1.07 ± 0.08
	Amplitude medians (across all frequencies)	*√|PPC| increase* *√|PPC| decrease* *FR increase (Hz)* *FR decrease (Hz)*	0.27 ± 0.06 -0.20 ± 0.03 0.79 ± 0.16 -0.91 ± 0.06	0.25 ± 0.06 -0.16 ± 0.04 0.76 ± 0.25 -0.90 ± 0.07	0.31 ± 0.06 -0.15 ± 0.03 0.78 ± 0.28 -0.91 ± 0.10	0.38 ± 0.05 -0.07 ± 0.02 0.98 ± 0.33 -0.95 ± 0.07	0.32 ± 0.03 -0.13 ± 0.02 0.85 ± 0.14 -0.91 ± 0.04

For each amplitude and frequency combination of sACS, the median and 95% confidence interval of firing rate and entrainment changes from baseline to stimulation for neurons which demonstrated significant changes in the respective firing pattern category (increased/decreased √|PPC|/FR) considered.

**Table 3.** Linear mixed effects model estimates of the influence of amplitude and frequency on entrainment.

Spike timing metric	Effects	Estimate	SE	df	t value	p	Significant
*√|PPC| increase*	(Intercept)	3.38E-01	5.12E-02	53.047	6.6083832	1.92E-08	***
	amplitude	-6.52E-05	1.59E-04	159.9039	-0.4096764	6.83E-01	
	frequency	-1.51E-03	1.82E-03	144.2337	-0.8271596	4.10E-01	
	amplitude:frequency	1.47E-05	6.00E-06	140.1434	2.4556188	1.53E-02	*
*√|PPC| decrease*	(Intercept)	-1.81E-01	2.68E-02	21.93016	-6.742407	9.07E-07	***
	amplitude	2.73E-04	1.03E-04	48.93921	2.6445035	1.10E-02	*
	frequency	-5.76E-04	1.44E-03	48.62915	-0.4004395	6.91E-01	
	amplitude:frequency	-3.42E-06	5.61E-06	49.88353	-0.6090954	5.45E-01	
FR increase	(Intercept)	9.33E-01	2.10E-01	26.19403	4.442814	0.000144181	***
	amplitude	5.40E-04	6.89E-04	178.91853	0.7833717	0.434444599	
	frequency	6.73E-03	8.12E-03	188.30817	0.8287981	0.408267938	
	amplitude:frequency	2.45E-05	3.34E-05	177.73474	0.7347547	0.463457231	
FR decrease	(Intercept)	-1.254082398	2.88E-01	6.81948	-4.3554	3.55E-03	**
	amplitude	-0.0004837166	1.67E-04	1766.53683	-2.897957	3.80E-03	**
	frequency	-0.0100800819	1.61E-03	1764.14498	-6.264056	4.70E-10	***
	amplitude:frequency	0.0000167024	6.72E-06	1761.87637	2.484652	1.31E-02	*

The dependence of the magnitude of entrainment or firing rate, that is, the change in Δ√|PPC| or ΔFR, on sACS frequency and amplitude when contrasting 1-minute sACS to 1 minute of baseline across neurons firing at ≥0.25 Hz found to be significant changes in spike timing (p = 0.05). The interactive effect of amplitude and frequency was found to significantly affect the degree of entrainment (*p = 0.02). Amplitude was found to have a significant effect on the magnitude by which entrainment was reduced (*p = 0.01). Amplitude and frequency were found to have both an independent and interactive significant effect on the decrease in FR (**p = 0.004, ***p < 0.0001, *p = 0.01).

Under sham sACS, no neurons showed significant changes in firing rate across any of the 1-minute baseline to sham stimulation paradigms (Supplementary Table S3.1). One or two neurons were spuriously detected as having significantly changed entrainment levels from the preceding minute of stimulation in some sham stimulation paradigms, without reflecting frequency and amplitude-specific trends reported under true sACS delivery (Supplementary Table S3.1).

### 3.2 Increasing sACS amplitude and frequency increased the degree of entrainment

We next examined the relationship between sACS amplitude and neuronal firing patterns. An LMER revealed that the interaction between amplitude and frequency positively affected the degree of entrainment (Table 3, p = 0.02; Fig. 6; Supplementary Tables S1.2–S1.5). Only amplitude was found to significantly affect decreases in entrainment (Table 3, p = 0.01; Fig. 7; Supplementary Tables S1.2–S1.5), with increasing amplitude yielding smaller magnitudes of entrainment decrease.

When considering linear regressions, with increasing sACS amplitudes, the degree of entrainment increased across 5 and 40 Hz stimulation frequencies (p = 0.004 and 0.0002, respectively); growing at a rate of 7.22 × 10^−4^ and 7.63 × 10^−4^ Δ√|PPC| per μA (Fig. 6B; Supplementary Table S1.3), and 40 Hz also showed an increase in the number of neurons entrained with increasing stimulation amplitude (6.04 × 10^−2^ % per μA, p = 0.007) (Fig. 6D; Supplementary Table S1.5). With increasing frequency, in contrast, increases in the degree of entrainment were seen at 400 μA sACS, at a rate of 5.10 × 10^−3^ Δ√|PPC| per Hz (p = 0.002) (Fig. 6A; Supplementary Table S1.4).

### 3.3 Higher frequencies of stimulation resulted in greater degrees of suppression

Neurons that increased their FR during sACS did not show a dependence of their induced FR change on the sACS parameters (Table 3). The majority of neurons responding to sACS decreased their FRs during stimulation (60% of neurons were inhibited by sACS while <10% were excited, Table 1), and an LMER revealed that amplitude and frequency had independent and interactive effects on the decrease in FR (Table 3, independent effect of amplitude: p = 0.004, independent effect of frequency: p < 0.0001, interactive effect of amplitude and frequency: p = 0.001).

Linear regressions of 100 μA sACS response data versus sACS frequency showed increases in the magnitude of FR increase in excited neurons with increasing frequency (3.65 × 10^−2^ ΔHz per Hz, p = 0.0003) (Fig. 8A; Supplementary Table S1.2). The 200 µA response regression showed a significant decrease in the percentage of excited neurons with increasing frequency (Fig. 8C; Supplementary Table S1.4) (-2.30 × 10^−3^ % per Hz, p = 0.01). The 5 Hz response regression across amplitudes (Fig. 8B; Supplementary Table S1.3) showed an increase in the magnitude of FR increase of excited neurons with increasing amplitude (2.22 × 10^−3^ ΔHz per μA, p = 0.002).

The responses to 50 and 10 μA sACS of suppressed neurons showed further reductions in FR with increasing frequency, at rates of -8.33 × 10^−3^ and -1.19 × 10^−2^ ΔHz per Hz (p = 0.0004 and <0.0001, respectively; Fig. 9A; Supplementary Table S1.2), with no (linear) stimulation frequency dependence of the fraction of neurons affected (Fig. 9C; Supplementary Table S1.4). Only in the 5 Hz sACS regression across amplitudes, FRs were further decreased with increasing amplitude, at a rate of -1.36 × 10^−3^ ΔHz per µA (p = 0.0008; Fig. 9B; Supplementary Table S1.3). However, the fraction of neurons affected was maintained across sACS amplitudes (Fig. 9D; Supplementary Table S1.5). When considering grouped FR changes, most amplitudes of stimulation elicited further suppression with increasing stimulation frequency (at rates of -1.02 × 10^−2^, -1.33 × 10^−2^, and -1.47 × 10^−2^ ΔHz per Hz for 50, 100, and 200 µA trials; p = 0.0003, <0.0001, and 0.0001, respectively; Fig. 10A; Supplementary Table S1.2), whereas the degree of suppression was largely maintained across amplitudes (Fig. 10B; Supplementary Table S1.3).

### 3.4 Long duration (20-minute) sACS experiments

To investigate sustained effects persisting beyond the offset of stimulation, we delivered 20-minute sACS at 40 Hz, chosen for its effectiveness in entraining neurons in the 1-minute paradigms. Stimulation was delivered at 100 μA, a tolerable amplitude for 20-minute sACS paradigms in awake rats (Yu et al., 2015) that is also substantially more effective than 50 μA (Baba et al., 2009; Vöröslakos et al., 2018; Wachter et al., 2011). The median electric field produced for these experiments was 1.65 ± 0.21 mV/mm (Fig. 1D).

A median of 44.00 ± 10.36 neurons were detected per rat, with 4.00 ± 0.60 neurons per electrode channel. Of these neurons, 51.61 ± 10.62% (or, cumulatively, 343 of 620) had FRs ≥0.25 Hz in at least half of all 1-minute windows across baseline and stimulation periods and were thus included in subsequent analysis. The median baseline firing rate and entrainment at baseline were 1.05 ± 4.92 Hz (Supplementary Fig. S1.1) and 0.14 ± 0.06 to 40 Hz frequencies (Supplementary Fig. S1.2).

### 3.5 During 20-minute sACS, neurons were on the whole excited

To understand the effects of prolonged stimulation, we compared changes in firing patterns from baseline to the entire 20-minute sACS period: 4% of all neurons were entrained, while in 2% of all neurons √|PPC| decreased (Table 4; Supplementary Fig. S2.1). When considering the FRs of neurons, 54% showed increased FRs, while 34% decreased their FRs (Table 4; Supplementary Fig. S2.2).

**Table 4.** Percentages of neurons affected across detection windows of the 20-minute sACS stimulation paradigm.

			Neurons affected per rat(%)	Neurons affected across all rats (343 neurons)	Changes in firing patterns of affected neurons per rat	Change in firing patterns across all (343) affected neurons
Detection window	20-minute stimulation	*Any change* *√|PPC| increase* *√|PPC| decrease* *FR increase* *FR decrease*	91.89 ± 5.10 0.00 ± 3.93 0.00 ± 1.27 63.41 ± 17.85 24.39 ± 15.14	88.63 3.79 2.04 54.23 33.53	0.04 ± 0.01 -0.07 ± 0.03 1.13 ± 0.67 Hz -0.34 ± 0.87 Hz	0.04 ± 0.01 -0.07 ± 0.03 0.91 ± 0.42 Hz -0.30 ± 0.37 Hz
	10-minute post-stimulation	*Any change* *√|PPC| increase* *√|PPC| decrease* *FR increase* *FR decrease*	94.12 ± 5.74 0.00 ± 1.39 2.44 ± 4.19 22.22 ± 21.35 48.78 ± 19.94	90.38 2.04 4.37 39.07 50.73	0.08 ± 0.09 -0.04 ± 0.05 0.80 ± 1.67 Hz -0.48 ± 1.22 Hz	0.07 ± 0.08 -0.04 ± 0.03 1.53 ± 1.41 Hz -0.43 ± 0.44 Hz

For each window considered (1-minute during stimulation, 20-minute during stimulation, 10-minute post-stimulation), the median and 95% confidence interval of the percentage of neurons per rat were detected as being significantly affected relative to all neurons detected in that trial that fired at ≥0.25 Hz, as well as the percentage affected across neurons from all rats.

### 3.6 Neuronal entrainment persisted in the 10 minutes following 20-minute sACS offset

To address the persistence of sACS effects, we contrasted the first 10 minutes following stimulation offset to the 10 minutes immediately preceding stimulation: 2% of all neurons maintained imposed spiking patterns (i.e., showed increases in entrainment from baseline *after* stimulus offset) (Table 4; Supplementary Fig. S2.3). In 4% of all neurons, the entrainment was *decreased* relative to pre-stimulus baseline (Table 3; Supplementary Fig. S2.1). Finally, half of all neurons (51%) had decreased firing post-stimulation (Table 4; Supplementary Fig. S2.4), while 39% showed increased FRs post-sACS (Table 4; Supplementary Fig. S2.4): excitation during sACS thus turned to suppression post-sACS.

### 3.7 Power increased near excited neurons, and decreased near suppressed and entrained neurons

Changes in the LFPs and LF-HF PAC reported on the effect of sACS on population dynamics. Since the large artifact produced by the stimulating waveform cannot be fully removed from low-frequency electrophysiological signals of interest, we could assess neither power nor LF-HF PAC during stimulation; instead, we examined the LFP in the 2 minutes immediately preceding and following sACS in electrode channels in which significantly affected neurons (i.e., those showing either √|PPC| or FR changes) had been detected in the MUA band analyses.

**Fig. 11.**
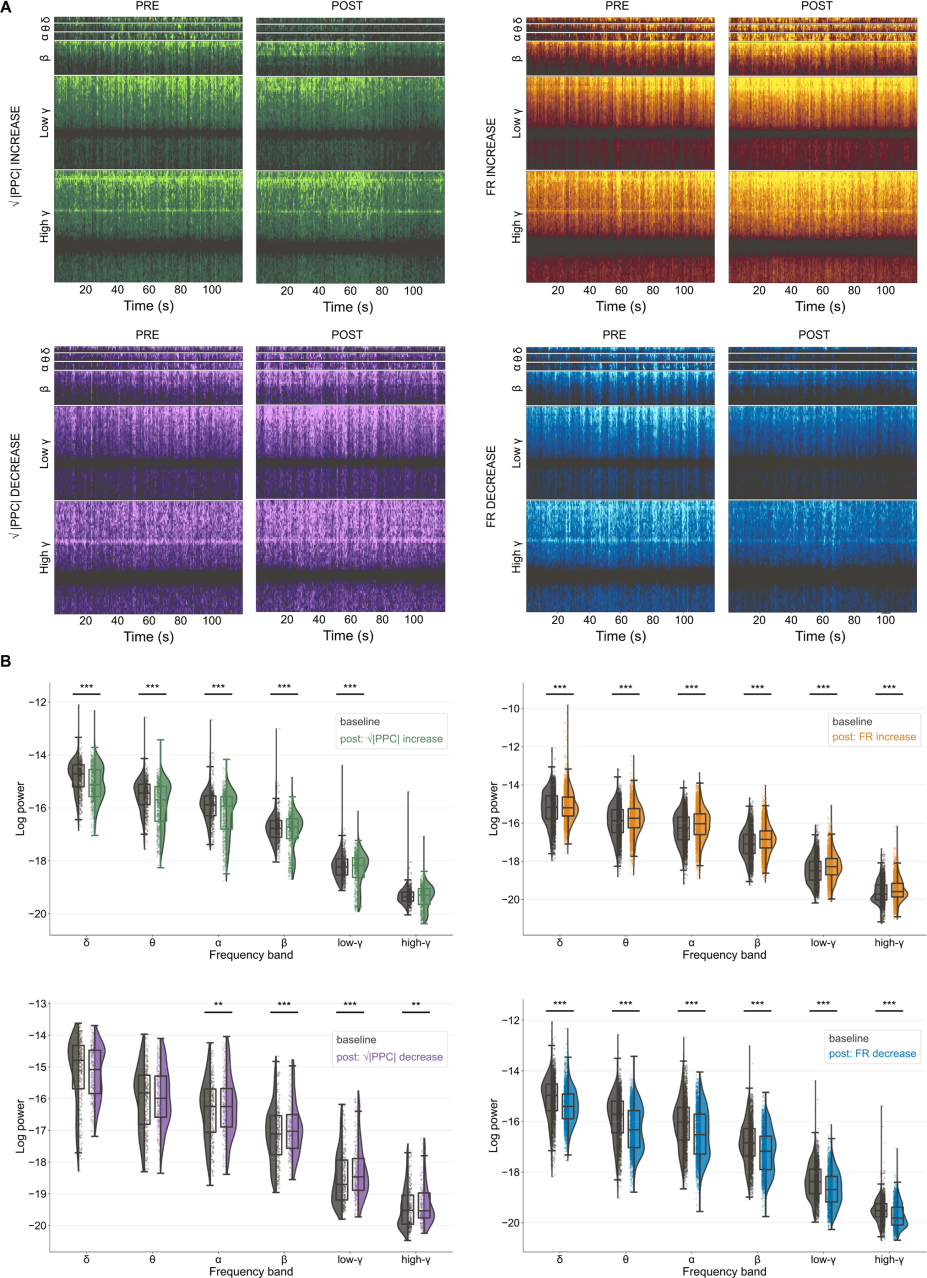
Frequency band power changes from the 2 minutes preceding to following 20-minute sACS were increased on electrode channels near excited neurons or neurons with reduced entrainment and decreased on channels proximal to suppressed or entrained neurons. Spectrograms showing the mean log power across frequency bands across the 2 minutes preceding and following 20-minute sACS, computed over 2s windows, for channels proximal to neurons which showed significantly increased entrainment (green, n = 12 channels), decreased entrainment (purple, n = 6 channels), increased firing rates (yellow, n = 78 channels), and decreased firing rates (blue, n = 47 channels) over the 20-minute sACS (A). The spectrograms visualize the signal over time, with changes in activity observed throughout the 2-minute analysis window. Raincloud plots showing the pairwise *LMER* comparison of the log power between pre- and post-sACS conditions (B). For channels near entrained neurons, significant decreases in log power observed across δ, θ, α, β, and low-γ frequency bands (emmean = -0.12, -0.15, -0,13, -0.06, and -0.04, SE = 0.01, 0.01, 0.01, 0.01, and 0.01, p = ***<0.0001, ***<0.0001, ***<0.0001, ***<0.0001, and ***0.0002, respectively), whereas near decreases in √|PPC|, channels showed increases in the log power of α, β, low-γ, and high-γ frequency bands (emmean = 0.05, 0.09, 0.09, and 0.09, SE = 0.02, 0.02, 0.02, and 0.02, p = **0.001, ***<0.0001, ***<0.0001, and **0.0007, respectively). Channels near excited neurons had increases in log power across all frequency bands (emmean = 0.04, 0.08, 0.10, 0.10, 0.09, and 0.05, SE = 0.00, 0.00, 0.005, 0.00, 0.00, and 0.00, p = ***<0.0001, ***<0.0001, ***<0.0001, ***<0.0001, ***<0.0001, and ***<0.0001, for δ, θ, α, β, low-γ, and high-γ, respectively), and near suppressed neurons, decreases across all bands (emmean = -0.14, -0.19, -0.18, -0.16, -0.13, and 0.09, SE = 0.01, 0.01, 0.01, 0.01, 0.01, and 0.01, p = ***<0.0001, ***<0.0001, ***<0.0001, ***<0.0001, ***<0.0001, and ***<0.0001, for δ, θ, α, β, low-γ, and high-γ, respectively).

Voltage levels of the 12 channels close to entrained neurons decreased in the log power across most frequency bands (excluding high-γ) from baseline to post-stimulus (Fig. 11; Supplementary Table S2.1). Voltage variations in the six channels near neurons with decreased entrainment showed increases in the log power of α, β, low-γ, and high-γ frequency bands (Fig. 11; Supplementary Table S2.1).

Increases in the log power were observed across frequency bands in the 78 channels associated with neurons whose FRs increased in response to sACS (Fig. 11; Supplementary Table S2.1). Conversely, the 47 channels proximal to neurons whose FRs decreased showed decrease in log power across frequency bands (Fig. 11; Supplementary Table S2.1).

In 80-minute LFP recordings under propofol anesthesia without sACS perturbation, power was maintained at 1.71E-06 ± 6.94E-07 mV/Hz, 9.70E-07 ± 3.89E-07 mV/Hz, 6.66E-07 ± 2.72E-07 mV/Hz, 3.03E-07 ± 9.35E-08, 8.40E-08 ± 1.74E-08 mV/Hz, and 3.61E-08 ± 3.50E-09 mV/Hz for δ, θ, α, β, low-γ, and high-γ band activity, respectively, with an average of 5 discrete high-amplitude events observed across all frequency bands of interest.

### 3.8 Close to excited neurons, LF-HF PAC increased, but decreased near suppressed and entrained neurons

**Fig. 12.**
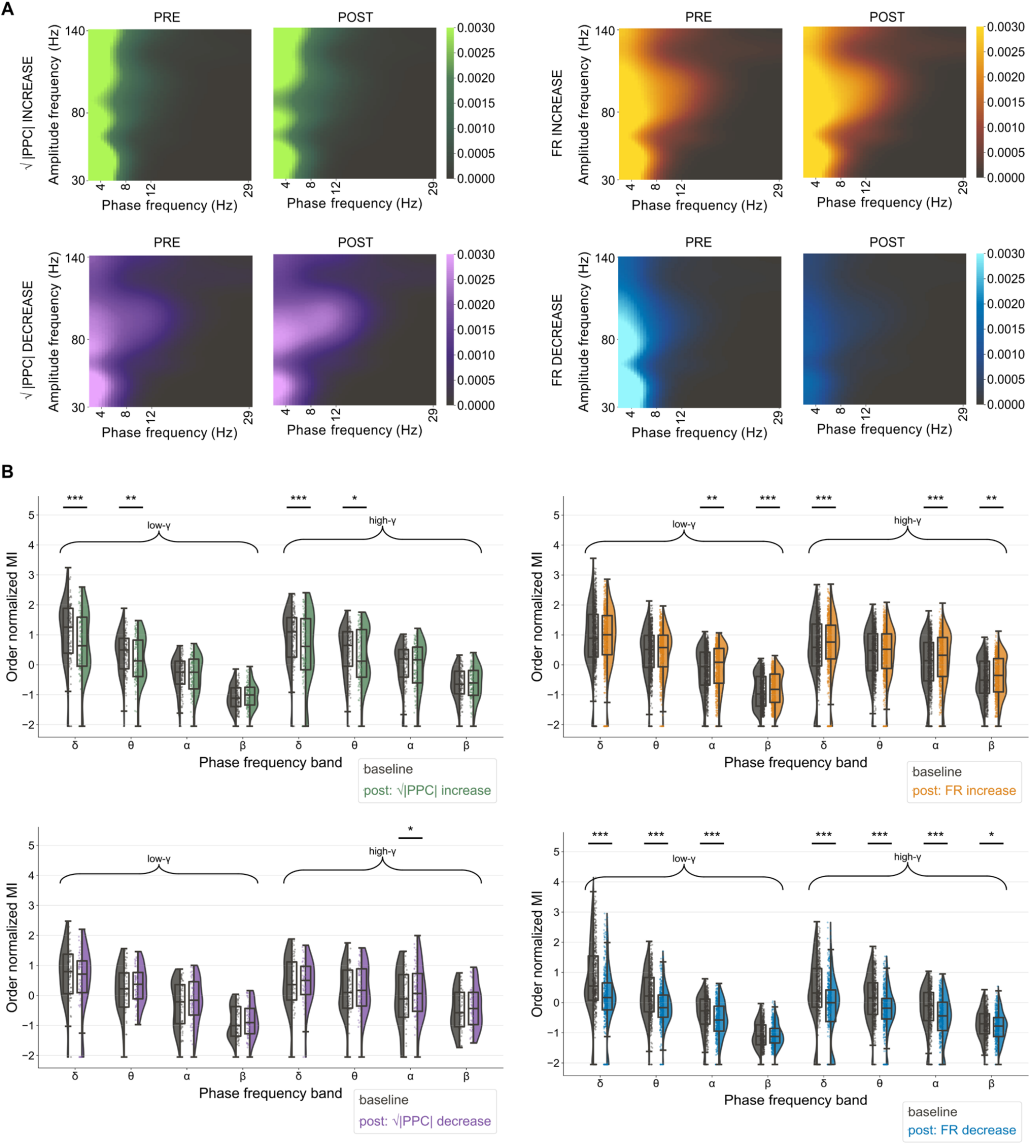
Phase-amplitude coupling changes from the 2 minutes preceding to following 20-minute sACS were increased in electrode channels near excited neurons or neurons with reduced entrainment and decreased in channels proximal to suppressed or entrained neurons. Comodulogram showing the average LF-HF PAC between frequency bands in the 2 minutes preceding and following 20-minute sACS, computed over 10s windows, for channels proximal to neurons which showed increased entrainment (green, n = 12 channels), decreased entrainment (purple, n = 6 channels), increased firing rates (yellow, n = 78 channels), and decreased firing rates (blue, n = 47 channels) over the 20 minutes of sACS (A). The comodulograms illustrate the variability of PAC within frequency bands (a broad range of frequencies contribute to the β, low-γ, and high-γ bands). Raincloud plots showing the pairwise *LMER* comparison of the order normalized MI between pre- and post-sACS conditions (B). For channels near entrained neurons, there was a significant decrease in the order normalized MI observed in δ to low-γ, θ to low-γ, δ to high-γ, and θ to high-γ (emmean = -0.39, -0.21, -0.15, and -0.15, SE = 0.07, 0.07, 0.07, and 0.07, p = ***<0.0001, **0.002, ***<0.0001, and *0.03, respectively), whereas channels near decreases in √|PPC| showed a significant increase in α to high-γ coupling (emmean = 0.22, SE = 0.10, p = *0.02). Channels near excited neurons showed increases in the order normalized MI between α to low-γ, β to low-γ, δ to high-γ, α to high-γ, and β to high-γ band pairs (emmean = 0.10, 0.10, 0.15, 0.12, and 0.09, SE = 0.03, 0.03, 0.03, 0.03, and 0.03, p = **0.0007, ***0.0005, ***<0.0001, ***<0.0001, and **0.002, respectively), and near suppressed neurons, decreases in δ to low-γ, θ to low-γ, α to low-γ, δ to high-γ, θ to high-γ, α to high-γ, and β to high-γ band pairs (emmean = -0.60, -0.37, -0.21, -0.54, -0.36, -0.29, and -0.06, SE = 0.04, 0.04, 0.04, 0.04, 0.04, 0.04, and 0.04, p = ***<0.0001, ***<0.0001, ***<0.0001, ***<0.0001, ***<0.0001, ***<0.0001, and *0.01, respectively).

Channels close to entrained neurons showed a significant decrease, from baseline to post-stimulus, in the order normalized MI of δ to low-γ, θ to low-γ, δ to high-γ, and θ to high-γ (Fig. 12; Supplementary Table S2.2). Channels close to neurons exhibiting decreases in √|PPC| showed a significant increase in the α to high-γ order normalized MIs (Fig. 12; Supplementary Table S2.2).

Increases in the order normalized MIs were observed across several band pairings on channels linked to increases in FRs (α to low-γ, β to low-γ, δ to high-γ, α to high-γ, and β to high-γ; Fig. 12; Supplementary Table S2.2), and decreases in the order normalized MI in channels alongside decreases in FR (δ to low-γ, θ to low-γ, α to low-γ, δ to high-γ, θ to high-γ, α to high-γ, and β to high-γ; Fig. 12; Supplementary Table S2.2). Overall, while changes in √|PPC| were observed alongside changes in lower frequency–phase coupling (δ/θ), changes in FR were observed alongside a broader set of LF-HF PAC changes that included the coupling of higher frequency phases (i.e., α and β) to high-frequency amplitudes.

Under propofol anesthesia without the delivery of electric stimulation, modulation indices averaged 1.17E-03 ± 1.11E-03, 3.88E-04 ± 3.86E-04, 3.90E-04 ± 3.48E-04, 2.04E-04 ± 1.87E-04, 7.79E-05 ± 8.31E-05, 1.09E-04 ± 1.12E-04, 1.06E-05 ± 9.64E-06, and 3.78E-05 ± 3.05E-05 for δ to low-γ, δ to high-γ, θ to low-γ, θ to high-γ, α to low-γ, α to high-γ, β to low-γ, and β to high-γ band couplings, respectively, across 80-minute recordings.

## 4 Discussion

Optimizing the ACS protocol in the clinic is difficult due to the limited sensitivity of non-invasive technologies for recording neuronal function. Furthermore, *in silico* simulations of neuronal responses to ACS, which are commonly used to fill the gaps in our understanding of neuromodulatory interventions effects, are confounded by the lack of ground truth experimental observations. The current study addressed this gap through a finely controlled experimental model study, leveraging intracortical multi-electrode arrays for high sensitivity recording of neuronal activity with controlled light sedation in co-housed littermates, to limit baseline variability.

### 4.1 sACS parameter exploration

Neuronal responses in our experiments showed dose dependence, in line with prior work (Johnson et al., 2020; Krause et al., 2022), with higher currents inducing more neurons to synchronize their firing to the stimulating waveform as well as increasing the degree of synchronization in their firing. A progressive suppression of firing rates was induced with increasing sACS frequency and amplitude. Alterations of FRs, largely within 2 Hz, were split between increases and decreases, in support of maintenance of the excitation/inhibition balance under external perturbations. The greater reductions in FR observed with increasing sACS frequency suggest that higher sACS frequencies induce more suppression, likely due to the recruitment of faster firing interneuronal populations (Fellous et al., 2001; X. Huang et al., 2024; Lee et al., 2024; Povysheva et al., 2005).

Since the temporal window during which sACS is either maximally de- or hyper-polarizing (dependent on an individual neuron’s orientation relative to the generated electric field) is broader at lower frequencies, low frequency sACS is expected to elicit less uniform phase synchronization, resulting in reduced Δ√|PPC| in comparison with that achieved at higher sACS frequencies (Asan et al., 2020). More synchronous firing promoted by endogenous gamma oscillations has been theorized as a mechanism of signal propagation (Jensen et al., 2014), as increased synchronicity in presynaptic inputs precipitates the action potentials in target neurons, thereby inducing a feed-forward drive (Jensen et al., 2007). The integration of postsynaptic potentials may also contribute to the increased number of entrained neurons at higher stimulation frequencies. Notwithstanding, keeping the overall level of synchronization enhancement modest prevents epileptiform activity that arises alongside excessive synchronization (Grasse et al., 2013; Ren et al., 2021). Lower frequencies, given their wider temporal windows, yield more varied spike times, reducing synchrony of activity and signal propagation (Asan et al., 2020; Fries, 2009; Jensen et al., 2007, 2014). Our findings thus support the potential of ACS to evoke tailored effects based on the frequency of the endogenous oscillatory activity (Jensen & Mazaheri, 2010): specifically, the delivery of lower frequencies of stimulation (such as α-band) to task-irrelevant regions to disrupt activity, and higher frequencies (such as γ-band) to regions where potentiation of activity is desirable (Gonzalez-Perez et al., 2019; Grover et al., 2022; Guerra, Suppa, et al., 2018; Herring et al., 2019; Hopfinger et al., 2017; Hutchinson et al., 2020; Klink et al., 2020; Schuhmann et al., 2022; Wöstmann et al., 2018).

Some neurons exhibited reduced entrainment during sACS, particularly at low amplitudes of stimulation, supporting the notion that stimulating waveforms compete with endogenous activity (Krause et al., 2022; Zhao et al., 2023). This idea is further supported by the higher incidence of reduced entrainment observed under weaker stimulation amplitudes, though the limited number of neurons displaying this diminution made the investigation of the amplitude dependence of this phenomenon challenging. Consideration of phase of the ongoing endogenous activity in addition to tailoring of the frequency, amplitude, and location of applied electric fields may produce further potentiation of the brain responses.

### 4.2 Effects of 20-minute sACS on firing rate and entrainment

Data from our longer duration sACS experiments support the feasibility of inducing persistent or “offline” effects. A small percentage of neurons retain imposed rhythms of firing in the 10 minutes following stimulation offset, indicating temporal coordination of firing can be enhanced beyond the sACS offset. The percentage of neurons showing *reduced* entrainment increased in the 10 minutes following stimulation offset. In line with the notion of competition between endogenous oscillatory activity and the applied stimulation waveform, wherein decreased entrainment values represent a shift in the preferred phase of firing (Krause et al., 2022), the increase in neurons with reduced entrainment following sACS offset may reflect a homeostatic process, whereby spike timing shifts back to synchronize with endogenous activity. The 20-minute sACS stimulation was on the whole excitatory: about a third of neurons decreased their FRs, while a half increased their FRs. However, in the 10 minutes post-stimulation, activity was predominantly subdued, with half of the neurons inhibited and 40% excited. FR changes remained within 2 Hz, similarly to FR changes prompted during 1-minute sACS trials.

### 4.3 Effects of 20-minute sACS on power and LF-HF PAC

The LFP is believed to be reflective of transmembrane synaptic currents and the balance of excitatory and inhibitory activity (Brunel & Wang, 2003), thereby indirectly reporting on the firing patterns of neuronal *ensembles* (Manning et al., 2009). Increases in individual neurons’ entrainment were associated with decreased log power across frequency bands. Decreases in entrainment of individual neurons, in turn, were associated with an increase in log power activity. Though increases in synchrony of firing are associated with increases in power (Buzsáki et al., 2012), particularly in high-frequency bands, the changes observed here result from the bifurcation of neuronal synchronization to either sACS waveform or endogenous oscillations. Log power increased in all frequency bands across channels close to excited neurons, and decreased in all bands across channels proximal to inhibited neurons. These associations were expected as increased firing activity is linked to wide-band power increases (Buzsáki et al., 2012).

It has been proposed that neural oscillations serve to aid in spatiotemporal organization across large scale brain networks, with low frequencies modulating excitability of neurons and consequently showing a phase association with the amplitude of fast (high-frequency) oscillations (Kim et al., 2022), which may be associated with neuronal firing and/or signal integration (Belitski et al., 2008; Einevoll et al., 2013; Gieselmann & Thiele, 2008). Consequently, LF-HF PAC has been interpreted as a mode of organization, like storing items on shelves, segregating the information encoded in high frequencies so as to facilitate neuronal computation (Alekseichuk et al., 2016). Changes in LF-HF PAC have been reported in the progression of brain pathologies: for example, increases in β to γ PAC are associated with increased symptoms severity in Parkinson’s disease patients (de Hemptinne et al., n.d.; Gong et al., 2021; Tsiokos et al., 2017; Yin et al., 2022), while increases in θ to γ PAC predict improvements in motor function over stroke recovery (Rustamov et al., 2022).

We found that channels proximal to entrained neurons showed decreases in LF-HF PAC. Given that the applied sACS waveforms can compete with ongoing oscillatory activity (Krause et al., 2022), altering neuronal spike timing and thereby affecting local field potentials, it is unsurprising that sACS interferes with the coupling between oscillatory bands. Increases in coupling are expected when delivering sACS synchronized to the phase of endogenous activity, or upon the delivery of a cross-frequency coupled ACS paradigm (Kim et al., 2022; Park et al., 2023). Channels on which excited neurons were detected showed a general increase in coupling, and those close to suppressed neurons, decreases in LF-HF PAC. Increased firing rates in association with elevated LF-HF PAC have been reported (Meidahl et al., 2019; Tamura et al., 2017) and were anticipated as increased organization or synchrony of activity yields larger voltage dynamics, thereby increasing the likelihood of depolarizing cells and triggering action potentials.

### 4.4 Electric field estimates

In humans, the maximum electric fields produced via tACS are estimated to reach maximal magnitudes of 0.5 mV/mm per 1 mA of stimulation (Guidetti et al., 2022; Opitz et al., 2016), though experimental measurements are confounded by the invasiveness of intracerebral recording electrodes. At the current safety threshold of 2 mA for clinical tACS, we anticipate neuromodulatory effects would approach those observed at amplitudes of 50 µA in current experiments. Given the marginally unfavorable outcomes reported in human studies when delivering currents above 2 mA (up to 4–6 mA; Chhatbar et al., 2017; Khadka et al., 2020; Workman et al., 2019), as well as the potential to increase electric field magnitudes by leveraging multi-electrode montages as shown in pre-clinical studies (Alekseichuk et al., 2019), we expect the greater neuronal modulation effects observed at the higher stimulation amplitudes in the current study to be clinically attainable in the future.

On a methodological note, while anesthesia significantly attenuates the neuronal excitability (specifically propofol, a GABA_A_ agonist, potentiates conductances from fast spiking to pyramidal cells, thereby inhibiting firing activity (Bieda & MacIver, 2004; Hanrahan et al., 2013; Kobayashi & Oi, 2017), particularly in the first 30 minutes following induction (Bastos et al., 2021)) and has been shown to reduce neuronal responsiveness to applied electric fields (Alekseichuk et al., 2022; Arena et al., 2021), the use of anesthesia is invaluable for acquiring high fidelity electrophysiological recordings over extended acquisition periods and results in significant stabilization of the baseline, which simplifies data interpretation (Breshears et al., 2010; Paasonen et al., 2018). Propofol anesthetic was selected as it better reflects awake state functional connectivity than do other anesthetics, such as isoflurane, medetomidine, and α-chloralose (Paasonen et al., 2018), while maintaining θ-γ coupling dynamics (Breshears et al., 2010). Notwithstanding, the shifting of neuronal oscillations toward lower (δ) frequencies under propofol anesthesia (Bastos et al., 2021; Reed & Plourde, 2015) and the corresponding reduction of network synchrony at higher frequencies may have reduced the responsiveness to sACS frequencies tested in this study. Future work should investigate parameter effect variability in awake animals, both in resting conditions and when completing tasks. While the lower minimum effective dose in the awake state potentiates neuronal responsiveness to electric stimulation, we expect greater competition between endogenous oscillatory activity and the sACS waveform in the awake state; phase asynchrony between the two waveforms can yield decreases in spike entrainment when neurons with firing locked to a preferred phase of endogenous oscillations shift to firing in relation to the sACS waveform (Krause et al., 2022). This competition may thus interfere with task-related activity, such as reach-to-grasp, improving or impairing performance contingent on sACS phase alignment to endogenous oscillations (Khanna et al., 2021). Online electrophysiological readouts to strategically synchronize sACS delivery with endogenous activity are needed to maximize the induction of constructive interference (when seeking to promote coordination of neuronal activity and boost synaptic connectivity) or destructive interference (to mitigate overly synchronous or task-irrelevant activity).

## 5 Conclusions

To our knowledge, this is the first comprehensive characterization of the neuronal effects of ACS parameters in the cortex: considering the degree of entrainment/firing rate modulation, the number of neurons affected, as well as the offline local network changes proximal to affected neurons. The transient imposition of novel patterns of synchrony in subsets of neurons, extending to altered activity across broader neuronal networks, supports the potential of ACS to induce localized changes in synaptic plasticity, pertinent given that synaptic vulnerability and decline of synaptic diversity are early drivers in neurodegenerative disease. The sensitivity of the brain response on sACS parameters described here can be used to impose a range of different effects on the brain region stimulated, and its downstream targets. High frequencies may be used to enhance synchrony of neuronal firing, potentiating signal propagation and supporting synaptic restructuring, and may have clinical relevance in diseases hallmarked by diminished neural connectivity (e.g., targeted to regions of the brain affected by lesions, such as in stroke, traumatic brain injury, or Parkinson’s disease). Low frequencies, in turn, can be used to broaden distributions in spike timing, and may have therapeutic use when delivered to interfere and attenuate with aberrant synchronous (e.g., in epilepsy) or task-irrelevant activity. Increasing stimulation magnitudes will increase the number of neurons affected as well as the extent of spike timing modulation, and can ergo be manipulated for localized effects with targeted electrode placement. Our results provide a basis for new understanding of how parameterization of alternating current stimulation can be leveraged to induce specific brain responses, providing a structured framework for the development of personalized neuromodulatory approaches for region and state-dependent restoration of brain function.

## Supplementary Material

Supplementary Material

## Data Availability

The data acquired in these experiments, along with the analysis scripts, are available through the Science Data Bank repository at https://doi.org/10.57760/sciencedb.32375. The analysis scripts used to analyze data in this study are openly available on GitHub at https://github.com/m-bellvila/Parametrizing-alternating-current-stimulation-for-neuromodulation.git.
