# Supplementary material for: Parametrizing alternating current stimulation for neuromodulation
Source: Imaging Neurosci (Camb). 2026 Jan 7;4:IMAG.a.1066. doi: 10.1162/IMAG.a.1066 (PMC12779756; doi:10.1162/IMAG.a.1066)
Supplement: Supplementary Material [file IMAG.a.1066_supp.pdf]

Supplemental

S1 *1-min sACS responses across frequency and amplitude*

Spike timing metric	Amplitude (μA)	Frequency (Hz)	eff_size	95% CI	emmean	SE of emmean	p	Signif
√|*PPC*| *increase*	**50**	**5**	1.10	1.05	1.63E-01	7.89E-02	4.03E-02	*
	**100**	**5**	1.32	1.05	1.96E-01	7.89E-02	1.38E-02	*
	**200**	**5**	1.97	8.04E-01	2.91E-01	6.03E-02	2.41E-06	***
	**400**	**5**	2.79	1.05	4.13E-01	7.89E-02	3.58E-07	***
	**50**	**10**	2.68	8.04E-01	3.96E-01	6.03E-02	2.73E-10	***
	**100**	**10**	2.71	8.80E-01	4.00E-01	6.61E-02	4.70E-09	***
	**200**	**10**	2.680	6.58E-01	3.96E-01	4.92E-02	2.99E-14	***
	**400**	**10**	2.470	5.50E-01	3.65E-01	4.10E-02	9.94E-17	***
	**50**	**20**	2.08	9.28E-01	3.08E-01	6.96E-02	1.44E-05	***
	**100**	**20**	2.27	1.14	3.36E-01	8.53E-02	1.06E-04	***
	**200**	**20**	1.93	8.04E-01	2.86E-01	6.03E-02	3.54E-06	***
	**400**	**20**	1.75	6.77E-01	2.58E-01	5.07E-02	6.65E-07	***
	**50**	**40**	1.64	1.24	2.43E-01	9.34E-02	9.86E-03	*
	**100**	**40**	1.55	8.81E-01	2.29E-01	6.61E-02	6.02E-04	**
	**200**	**40**	2.81	6.77E-01	4.14E-01	5.07E-02	1.27E-14	***
	**400**	**40**	3.45	4.03E-01	5.09E-01	2.95E-02	7.71E-45	***
√|*PPC*| *decrease*	**50**	**5**	-3.74	1.19	-1.70E-01	2.42E-02	1.39E-08	***
	**100**	**5**	-3.48	1.660	-1.58E-01	3.70E-02	1.10E-04	***
	**200**	**5**	-2.98	1.48	-1.35E-01	3.20E-02	1.31E-04	***
	**400**	**5**	-1.67	1.42	-7.56E-02	3.20E-02	2.29E-02	*
	**50**	**10**	-3.24	1.34	-1.47E-01	2.86E-02	6.89E-06	***
	**100**	**10**	-3.64	1.08	-1.65E-01	2.26E-02	5.50E-09	***
	**200**	**10**	-3.53	1.47	-1.60E-01	3.20E-02	1.09E-05	***
	**400**	**10**	-1.520	1.66	-6.88E-02	3.70E-02	6.96E-02	
	**50**	**20**	-3.84	1.64	-1.74E-01	3.70E-02	2.70E-05	***
	**100**	**20**	-1.68	2.03	-7.62E-02	4.53E-02	1.00E-01	
	**200**	**20**	-3.35	1.29	-1.52E-01	2.86E-02	4.02E-06	***
	**400**	**20**	*NA*	*NA*	*NA*	*NA*	*NA*	
	**50**	**40**	-4.51	2.86	-2.04E-01	6.41E-02	2.67E-03	**

	**100**	**40**	-5.97	2.84	-2.71E-01	6.41E-02	1.25E-04	***
	**200**	**40**	-4.15	2.00	-1.88E-01	4.53E-02	1.56E-04	***
	**400**	**40**	-2.930	2.02	-1.33E-01	4.53E-02	5.51E-03	*
FR increase	**50**	**5**	4.76E-01	6.83E-01	7.58E-01	5.17E-01	1.43E-01	
	**100**	**5**	4.74E-01	5.42E-01	7.55E-01	3.98E-01	5.88E-02	
	**200**	**5**	9.62E-01	6.41E-01	1.53	4.80E-01	1.57E-03	**
	**400**	**5**	8.70E-01	7.38E-01	1.39	5.63E-01	1.44E-02	*
	**50**	**10**	8.88E-01	1.01	1.41	7.96E-01	7.68E-02	
	**100**	**10**	5.52E-01	7.60E-01	8.79E-01	5.81E-01	1.32E-01	
	**200**	**10**	3.65E-01	8.08E-01	5.82E-01	6.25E-01	3.52E-01	
	**400**	**10**	8.44E-01	7.00E-01	1.34	5.31E-01	1.19E-02	*
	**50**	**20**	5.57E-01	9.59E-01	8.87E-01	7.51E-01	2.38E-01	
	**100**	**20**	1.27	9.61E-01	2.03	7.51E-01	7.33E-03	*
	**200**	**20**	7.73E-01	1.08	1.23	8.51E-01	1.49E-01	
	**400**	**20**	4.84E-01	1.270	7.71E-01	1.01	4.45E-01	
	**50**	**40**	5.77E-01	1.01	9.18E-01	7.96E-01	2.50E-01	
	**100**	**40**	1.17	9.13E-01	1.86	7.12E-01	9.37E-03	*
	**200**	**40**	6.49E-01	1.08	1.03	8.51E-01	2.25E-01	
	**400**	**40**	1.39	8.75E-01	2.21	6.79E-01	1.25E-03	**
FR decrease	**50**	**5**	-9.60E-01	2.36E-01	-9.54E-01	1.20E-01	1.49E-15	***
	**100**	**5**	-1.25	2.21E-01	-1.24	1.12E-01	1.75E-28	***
	**200**	**5**	-1.85	2.71E-01	-1.84	1.37E-01	4.98E-41	***
	**400**	**5**	-1.42	2.89E-01	-1.41	1.46E-01	6.36E-22	***
	**50**	**10**	-9.74E-01	2.64E-01	-9.67E-01	1.34E-01	5.06E-13	***
	**100**	**10**	-8.86E-01	2.63E-01	-8.80E-01	1.33E-01	4.11E-11	***
	**200**	**10**	-8.74E-01	2.19E-01	-8.68E-01	1.11E-01	5.57E-15	***
	**400**	**10**	-1.05	2.33E-01	-1.04	1.18E-01	1.25E-18	***
	**50**	**20**	-1.220	2.35E-01	-1.21	1.19E-01	3.45E-24	***
	**100**	**20**	-9.14E-01	2.87E-01	-9.07E-01	1.46E-01	4.68E-10	***
	**200**	**20**	-1.11	2.56E-01	-1.10	1.30E-01	2.21E-17	***
	**400**	**20**	-1.11	2.54E-01	-1.10	1.29E-01	9.46E-18	***
	**50**	**40**	-1.23	2.55E-01	-1.22	1.29E-01	2.93E-21	***
	**100**	**40**	-1.56	2.40E-01	-1.55	1.22E-01	5.26E-37	***
	**200**	**40**	-1.61	2.07E-01	-1.60	1.05E-01	2.98E-52	***

	**400**	**40**	-1.200	2.06E-01	-1.19	1.04E-01	5.18E-30	***

Table S1.1. Pairwise comparisons of spike timing metrics before and after 1-min sACS.
For all stimulation frequencies and amplitudes tested, pairwise comparisons of the LMER were used to report changes in spike timing metrics (changes in $\sqrt{|PPC|}$ or FR); the effect size and 95% confidence interval as well as the estimated marginal means, standard error of the estimated marginal means, and p-value.

Spike timing metric	Amplitude (µA)	Slope (B_1)	Intercept (B_0)	R^2	p	SE	Signif
√|***PPC***| ***increase***	**50**	-6.80E-04	3.10E-01	2.21E-03	7.95E-01	2.60E-03	
	**100**	-2.01E-03	3.33E-01	2.74E-02	3.57E-01	2.15E-03	
	**200**	2.16E-03	3.15E-01	1.68E-02	3.28E-01	2.19E-03	
	**400**	5.10E-03	2.88E-01	9.10E-02	2.29E-03	1.63E-03	**
√|***PPC***| ***decrease***	**50**	-9.61E-04	-1.54E-01	1.68E-02	6.32E-01	1.96E-03	
	**100**	-1.60E-03	-1.38E-01	4.42E-02	4.71E-01	2.14E-03	
	**200**	-1.23E-03	-1.34E-01	6.51E-02	3.59E-01	1.30E-03	
	**400**	-1.74E-03	-6.09E-02	4.69E-01	4.17E-02	6.99E-04	
FR increase	**50**	1.23E-03	9.14E-01	8.52E-04	8.51E-01	6.50E-03	
	**100**	3.65E-02	6.32E-01	1.84E-01	3.22E-04	9.60E-03	***
	**200**	-9.10E-03	1.29E+00	1.16E-02	4.60E-01	1.22E-02	
	**400**	2.34E-02	1.12E+00	7.07E-02	6.20E-02	1.22E-02	
FR decrease	**50**	-8.33E-03	-9.37E-01	2.46E-02	4.08E-04	2.34E-03	***
	**100**	-1.19E-02	-9.61E-01	4.04E-02	6.64E-06	2.61E-03	***
	**200**	-8.06E-03	-1.16E+00	9.28E-03	2.25E-02	3.52E-03	
	**400**	7.86E-04	-1.19E+00	2.04E-04	7.42E-01	2.38E-03	
Overall FR change	**50**	-1.02E-02	-7.43E-01	2.32E-02	3.40E-04	2.82E-03	***
	**100**	-1.33E-02	-6.71E-01	2.69E-02	9.55E-05	3.39E-03	***
	**200**	-1.47E-02	-8.33E-01	2.36E-02	1.38E-04	3.84E-03	***
	**400**	-3.28E-03	-8.72E-01	1.76E-03	3.12E-01	3.24E-03	

Table S1.2. Linear regressions of the degree of change in spike timing metric across frequencies.

For each stimulation amplitude and spike timing metric, the results from the linear regressions performed across frequencies assessing changes in spike timing or frequency.

Spike timing metric	Frequency (Hz)	Slope (B_1)	Intercept (B_0)	R^2	p	SE	Signif
√|PPC| increase	**5**	7.22E-04	1.32E-01	2.36E-01	4.12E-03	2.33E-04	**
	**10**	-1.06E-04	4.10E-01	4.75E-03	5.82E-01	1.92E-04	
	**20**	-1.73E-04	3.27E-01	2.21E-02	3.36E-01	1.77E-04	
	**40**	7.63E-04	2.10E-01	1.64E-01	1.59E-04	1.93E-04	***
√|PPC| decrease	**5**	2.67E-04	-1.84E-01	3.98E-01	5.01E-03	8.22E-05	*
	**10**	2.36E-04	-1.81E-01	1.82E-01	6.04E-02	1.18E-04	
	**20**	9.91E-06	-1.45E-01	6.90E-05	9.82E-01	4.22E-04	
	**40**	3.00E-04	-2.53E-01	6.06E-01	6.83E-02	1.21E-04	
FR increase	**5**	2.22E-03	6.88E-01	1.04E-01	2.01E-03	6.98E-04	**
	**10**	8.55E-04	8.56E-01	1.90E-02	3.20E-01	8.51E-04	
	**20**	-1.45E-03	1.52E+00	2.57E-02	3.98E-01	1.69E-03	
	**40**	2.64E-03	1.07E+00	7.67E-02	1.02E-01	1.57E-03	
FR decrease	**5**	-1.36E-03	-1.10E+00	2.27E-02	7.97E-04	4.02E-04	**
	**10**	-3.36E-04	-8.71E-01	6.60E-03	6.36E-02	1.81E-04	
	**20**	4.46E-05	-1.10E+00	7.22E-05	8.55E-01	2.43E-04	
	**40**	5.80E-04	-1.51E+00	6.16E-03	5.26E-02	2.98E-04	
Overall FR change	**5**	-7.51E-04	-8.32E-01	4.56E-03	1.04E-01	4.61E-04	
	**10**	-6.89E-05	-7.39E-01	1.25E-04	7.88E-01	2.57E-04	
	**20**	-2.33E-04	-9.09E-01	1.14E-03	4.52E-01	3.09E-04	
	**40**	6.31E-04	-1.36E+00	4.84E-03	7.69E-02	3.56E-04	

Table S1.3 Linear regressions of the degree of change in spike timing metric across amplitudes.

For each stimulation frequency and spike timing metric, the results from the linear regressions performed across amplitudes assessing changes in spike timing or frequency.

Spike timing metric	Amplitude (μA)	Slope (B_1)	Intercept (B_0)	R^2	p	SE	Signif
√|*PPC*| *increase*	**50**	-3.26E-05	5.91E-02	1.78E-05	9.80E-01	1.32E-03	
	**100**	1.27E-03	2.56E-02	5.27E-02	1.85E-01	9.40E-04	
	**200**	2.24E-03	4.65E-02	5.18E-02	1.82E-01	1.64E-03	
	**400**	4.58E-03	9.42E-02	7.10E-02	1.62E-01	3.19E-03	
√|*PPC*| *decrease*	**50**	-1.15E-03	4.77E-02	3.36E-02	2.85E-01	1.06E-03	
	**100**	-2.13E-04	2.19E-02	3.96E-03	7.19E-01	5.87E-04	
	**200**	-5.81E-05	1.75E-02	4.54E-04	9.02E-01	4.68E-04	
	**400**	-7.37E-04	3.03E-02	6.58E-02	1.79E-01	5.35E-04	
FR increase	**50**	-1.36E-03	1.05E-01	2.31E-02	3.76E-01	1.52E-03	
	**100**	-8.60E-04	1.28E-01	4.49E-03	7.02E-01	2.23E-03	
	**200**	-2.30E-03	1.05E-01	1.78E-01	1.05E-02	8.49E-04	*
	**400**	-1.48E-03	1.47E-01	1.76E-02	4.93E-01	2.13E-03	
FR decrease	**50**	1.18E-03	6.08E-01	5.26E-03	6.74E-01	2.77E-03	
	**100**	4.31E-03	5.26E-01	5.90E-02	1.60E-01	2.99E-03	
	**200**	5.20E-03	5.14E-01	9.66E-02	6.50E-02	2.73E-03	
	**400**	2.91E-03	5.97E-01	4.76E-02	2.56E-01	2.51E-03	

Table S1.4 Linear regressions of the percentage of neurons affected across frequencies.

For each stimulation amplitude and spike timing metric, the results from the linear regressions performed across frequencies assessing the percentage of neurons affected.

Spike timing metric	Frequency (Hz)	Slope (B_1)	Intercept (B_0)	R^2	p	SE	Signif
√|*PPC*| *increase*	5	2.00E-04	8.53E-03	9.38E-02	8.29E-02	1.12E-04	
	10	1.68E-04	5.67E-02	3.66E-02	2.71E-01	1.50E-04	
	20	5.09E-04	1.67E-02	1.14E-01	5.48E-02	2.55E-04	
	40	6.04E-04	1.57E-02	2.01E-01	6.86E-03	2.10E-04	*
√|*PPC*| *decrease*	5	7.36E-06	3.07E-02	2.01E-01	9.57E-01	1.36E-04	
	10	-3.77E-05	2.61E-02	2.01E-01	3.81E-01	4.25E-05	
	20	-5.31E-05	2.64E-02	2.01E-01	4.26E-01	6.58E-05	
	40	1.34E-06	9.05E-03	2.01E-01	9.65E-01	3.02E-05	
FR increase	5	1.04E-04	8.84E-02	2.53E-02	3.77E-01	1.16E-04	
	10	1.70E-04	8.46E-02	2.17E-02	3.98E-01	1.99E-04	
	20	-1.29E-04	1.05E-01	7.98E-03	6.21E-01	2.58E-04	
	40	1.06E-04	4.23E-02	2.18E-02	3.97E-01	1.24E-04	
FR decrease	5	8.78E-05	5.40E-01	2.38E-03	7.88E-01	3.23E-04	
	10	-4.69E-05	6.08E-01	9.32E-04	8.62E-01	2.68E-04	
	20	1.62E-04	6.25E-01	8.55E-03	6.09E-01	3.13E-04	
	40	1.25E-04	6.63E-01	6.25E-03	6.52E-01	2.74E-04	

Table S1.5 Linear regressions of the percentage of neurons affected across amplitudes.
For each stimulation frequency and spike timing metric, the results from the linear regressions performed across amplitudes assessing the percentage of neurons affected.

Spike timing metric	Variable	Sum Sq	Mean Sq	NumDF	F value	p
√|*PPC*| *increase*	**tp**	8.99E-22	8.99E-22	1	1.70E-20	1.00E+00
	**freqband**	1.61E+03	3.22E+02	5	6.10E+03	0.00E+00
	**amp**	6.13E+00	6.13E+00	1	1.16E+02	5.07E-27
	**freq**	2.31E+01	2.31E+01	1	4.38E+02	9.19E-97
	**tp:freqband**	1.61E-21	3.22E-22	5	6.10E-21	1.00E+00
	**tp:amp**	2.64E-21	2.64E-21	1	5.00E-20	1.00E+00
	**freqband:amp**	9.61E+00	1.92E+00	5	3.64E+01	2.46E-37
	**tp:freq**	1.25E-21	1.25E-21	1	2.37E-20	1.00E+00
	**freqband:freq**	3.60E+01	7.19E+00	5	1.36E+02	8.24E-144
	**amp:freq**	7.46E+00	7.46E+00	1	1.41E+02	1.60E-32
	**tp:freqband:amp**	4.76E-21	9.52E-22	5	1.80E-20	1.00E+00
	**tp:freqband:freq**	2.27E-21	4.55E-22	5	8.61E-21	1.00E+00
	**tp:amp:freq**	3.17E-21	3.17E-21	1	6.00E-20	1.00E+00
	**freqband:amp:freq**	2.88E+01	5.77E+00	5	1.09E+02	5.32E-115
	**tp:freqband:amp:freq**	5.85E-21	1.17E-21	5	2.21E-20	1.00E+00
√|*PPC*| *decrease*	**tp**	1.14E-22	1.14E-22	1	3.18E-21	1.00E+00
	**freqband**	4.92E+02	9.85E+01	5	2.75E+03	0.00E+00
	**amp**	1.47E+00	1.47E+00	1	4.10E+01	1.59E-10
	**freq**	8.31E-03	8.31E-03	1	2.32E-01	6.30E-01
	**tp:freqband**	1.56E-22	3.11E-23	5	8.70E-22	1.00E+00
	**tp:amp**	3.71E-23	3.71E-23	1	1.04E-21	1.00E+00
	**freqband:amp**	5.08E+00	1.02E+00	5	2.84E+01	9.66E-29
	**tp:freq**	1.12E-22	1.12E-22	1	3.13E-21	1.00E+00
	**freqband:freq**	6.23E+00	1.25E+00	5	3.48E+01	1.82E-35
	**amp:freq**	9.14E-03	9.14E-03	1	2.56E-01	6.13E-01
	**tp:freqband:amp**	4.11E-23	8.21E-24	5	2.29E-22	1.00E+00
	**tp:freqband:freq**	1.79E-22	3.58E-23	5	1.00E-21	1.00E+00
	**tp:amp:freq**	5.61E-23	5.61E-23	1	1.57E-21	1.00E+00
	**freqband:amp:freq**	3.08E+00	6.17E-01	5	1.72E+01	4.87E-17
	**tp:freqband:amp:freq**	8.17E-23	1.63E-23	5	4.57E-22	1.00E+00
FR increase	**tp**	8.04E-25	8.04E-25	1	1.23E-23	1.00E+00
	**freqband**	3.74E+02	7.48E+01	5	1.14E+03	0.00E+00
	**amp**	1.37E+02	1.37E+02	1	2.10E+03	0.00E+00
	**freq**	3.68E+00	3.68E+00	1	5.63E+01	6.67E-14
	**tp:freqband**	1.96E-22	3.92E-23	5	5.99E-22	1.00E+00

	**tp:amp**	1.83E-24	1.83E-24	1	2.80E-23	1.00E+00
	**freqband:amp**	2.67E+00	5.34E-01	5	8.16E+00	1.06E-07
	**tp:freq**	3.97E-26	3.97E-26	1	6.07E-25	1.00E+00
	**freqband:freq**	5.87E+00	1.17E+00	5	1.79E+01	9.10E-18
	**amp:freq**	1.24E+00	1.24E+00	1	1.90E+01	1.33E-05
	**tp:freqband:amp**	1.58E-22	3.17E-23	5	4.84E-22	1.00E+00
	**tp:freqband:freq**	1.82E-22	3.64E-23	5	5.57E-22	1.00E+00
	**tp:amp:freq**	6.54E-25	6.54E-25	1	1.00E-23	1.00E+00
	**freqband:amp:freq**	2.11E+00	4.23E-01	5	6.46E+00	5.26E-06
	**tp:freqband:amp:freq**	1.38E-22	2.76E-23	5	4.22E-22	1.00E+00
FR decrease	**tp**	3.62E-22	3.62E-22	1	8.38E-21	1.00E+00
	**freqband**	8.67E+03	1.73E+03	5	4.01E+04	0.00E+00
	**amp**	5.94E+01	5.94E+01	1	1.38E+03	4.16E-300
	**freq**	6.65E-01	6.65E-01	1	1.54E+01	8.80E-05
	**tp:freqband**	4.17E-21	8.35E-22	5	1.93E-20	1.00E+00
	**tp:amp**	3.58E-21	3.58E-21	1	8.29E-20	1.00E+00
	**freqband:amp**	9.63E+00	1.93E+00	5	4.46E+01	3.68E-46
	**tp:freq**	1.13E-20	1.13E-20	1	2.62E-19	1.00E+00
	**freqband:freq**	3.30E+00	6.61E-01	5	1.53E+01	4.63E-15
	**amp:freq**	2.11E-01	2.11E-01	1	4.89E+00	2.70E-02
	**tp:freqband:amp**	5.00E-20	1.00E-20	5	2.31E-19	1.00E+00
	**tp:freqband:freq**	2.66E-20	5.31E-21	5	1.23E-19	1.00E+00
	**tp:amp:freq**	3.74E-22	3.74E-22	1	8.66E-21	1.00E+00
	**freqband:amp:freq**	1.44E+00	2.89E-01	5	6.69E+00	3.08E-06
	**tp:freqband:amp:freq**	4.32E-21	8.64E-22	5	2.00E-20	1.00E+00

Table S1.6. Anova summary table of linear mixed effects model of power before and after 1-min sACS conditions.

For local field potentials on electrode channels proximal to neurons which demonstrated significant modulations in spike timing metrics (increase/decrease firing rate/entrainment), the anova summary table reporting the influence of variables on the log power before and after 1-min of sACS. Variables included in the LMER were timepoint (‘tp’: baseline or post-sACS), frequency band (‘freqband’: θ, α, β, low-γ, or high-γ), sACS amplitude (‘amp’: 50, 100, 200, or 400 μA), and sACS frequency (‘freq’: 5, 10, 20, or 40 Hz).

Spike timing metric	Variable	Sum Sq	Mean Sq	NumDF	F value	p
√|*PPC*| *increase*	**tp**	6.14E-02	6.14E-02	1	9.80E-02	7.54E-01
	**bandrel**	3.73E+02	7.45E+01	5	1.19E+02	1.42E-123
	**amp**	4.35E+00	4.35E+00	1	6.94E+00	8.44E-03
	**freq**	5.68E+00	5.68E+00	1	9.07E+00	2.60E-03
	**tp:bandrel**	6.79E-02	1.36E-02	5	2.17E-02	1.00E+00
	**tp:amp**	5.97E-02	5.97E-02	1	9.54E-02	7.57E-01
	**bandrel:amp**	1.34E+01	2.67E+00	5	4.27E+00	7.06E-04
	**tp:freq**	4.60E-02	4.60E-02	1	7.35E-02	7.86E-01
	**bandrel:freq**	2.40E+01	4.80E+00	5	7.66E+00	3.37E-07
	**amp:freq**	5.29E+00	5.29E+00	1	8.45E+00	3.66E-03
	**tp:bandrel:amp**	6.17E-02	1.23E-02	5	1.97E-02	1.00E+00
	**tp:bandrel:freq**	9.09E-02	1.82E-02	5	2.90E-02	1.00E+00
	**tp:amp:freq**	5.72E-02	5.72E-02	1	9.13E-02	7.62E-01
	**bandrel:amp:freq**	6.83E+00	1.37E+00	5	2.18E+00	5.32E-02
	**tp:bandrel:amp:freq**	6.73E-02	1.35E-02	5	2.15E-02	1.00E+00
√|*PPC*| *decrease*	**tp**	1.44E-03	1.44E-03	1	2.56E-03	9.60E-01
	**bandrel**	8.57E+01	1.71E+01	5	3.04E+01	2.21E-30
	**amp**	7.24E-03	7.24E-03	1	1.28E-02	9.10E-01
	**freq**	2.44E-01	2.44E-01	1	4.32E-01	5.11E-01
	**tp:bandrel**	7.40E-02	1.48E-02	5	2.63E-02	1.00E+00
	**tp:amp**	5.88E-03	5.88E-03	1	1.04E-02	9.19E-01
	**bandrel:amp**	3.72E+00	7.44E-01	5	1.32E+00	2.53E-01
	**tp:freq**	3.91E-04	3.91E-04	1	6.94E-04	9.79E-01
	**bandrel:freq**	3.00E+00	6.00E-01	5	1.06E+00	3.78E-01
	**amp:freq**	7.78E-02	7.78E-02	1	1.38E-01	7.10E-01
	**tp:bandrel:amp**	5.53E-02	1.11E-02	5	1.96E-02	1.00E+00
	**tp:bandrel:freq**	5.73E-02	1.15E-02	5	2.03E-02	1.00E+00
	**tp:amp:freq**	3.57E-03	3.57E-03	1	6.33E-03	9.37E-01
	**bandrel:amp:freq**	2.66E+00	5.32E-01	5	9.44E-01	4.51E-01
	**tp:bandrel:amp:freq**	2.45E-02	4.90E-03	5	8.70E-03	1.00E+00
FR increase	**tp**	3.57E-03	3.57E-03	1	5.87E-03	9.39E-01
	**bandrel**	4.70E+01	9.39E+00	5	1.54E+01	4.47E-15
	**amp**	1.10E+01	1.10E+01	1	1.81E+01	2.16E-05

	**freq**	3.30E-01	3.30E-01	1	5.43E-01	4.61E-01
	**tp:bandrel**	4.98E-02	9.96E-03	5	1.64E-02	1.00E+00
	**tp:amp**	8.08E-04	8.08E-04	1	1.33E-03	9.71E-01
	**bandrel:amp**	6.71E-01	1.34E-01	5	2.21E-01	9.54E-01
	**tp:freq**	2.10E-03	2.10E-03	1	3.46E-03	9.53E-01
	**bandrel:freq**	6.38E+00	1.28E+00	5	2.10E+00	6.25E-02
	**amp:freq**	3.81E-01	3.81E-01	1	6.27E-01	4.29E-01
	**tp:bandrel:amp**	1.09E-01	2.17E-02	5	3.57E-02	9.99E-01
	**tp:bandrel:freq**	1.29E-01	2.59E-02	5	4.26E-02	9.99E-01
	**tp:amp:freq**	9.52E-04	9.52E-04	1	1.56E-03	9.68E-01
	**bandrel:amp:freq**	1.41E+01	2.83E+00	5	4.65E+00	3.12E-04
	**tp:bandrel:amp:freq**	1.51E-01	3.03E-02	5	4.98E-02	9.98E-01
FR decrease	**tp**	8.41E-03	8.41E-03	1	1.46E-02	9.04E-01
	**bandrel**	1.89E+03	3.77E+02	5	6.54E+02	0.00E+00
	**amp**	7.35E+00	7.35E+00	1	1.28E+01	3.55E-04
	**freq**	1.27E+00	1.27E+00	1	2.21E+00	1.37E-01
	**tp:bandrel**	6.10E-03	1.22E-03	5	2.12E-03	1.00E+00
	**tp:amp**	7.96E-04	7.96E-04	1	1.38E-03	9.70E-01
	**bandrel:amp**	1.06E+01	2.12E+00	5	3.68E+00	2.49E-03
	**tp:freq**	5.45E-04	5.45E-04	1	9.46E-04	9.75E-01
	**bandrel:freq**	6.96E+00	1.39E+00	5	2.41E+00	3.38E-02
	**amp:freq**	7.45E-01	7.45E-01	1	1.29E+00	2.55E-01
	**tp:bandrel:amp**	1.54E-02	3.07E-03	5	5.33E-03	1.00E+00
	**tp:bandrel:freq**	3.26E-02	6.53E-03	5	1.13E-02	1.00E+00
	**tp:amp:freq**	2.32E-03	2.32E-03	1	4.03E-03	9.49E-01
	**bandrel:amp:freq**	5.93E+00	1.19E+00	5	2.06E+00	6.74E-02
	**tp:bandrel:amp:freq**	2.16E-02	4.32E-03	5	7.50E-03	1.00E+00

Table S1.7. Anova summary table of linear mixed effects model of phase-amplitude coupling before and after 1-min sACS conditions.

For local field potentials on electrode channels proximal to neurons which demonstrated significant modulations in spike timing metrics (increase/decrease firing rate/entrainment), the anova summary table reporting the influence of variables on the order normalized MI before and after 1-min of sACS. Variables included in the LMER were timepoint (‘tp’: baseline or post-sACS), frequency band relationships considered (‘bandrel’: θ to low-γ, α to low-γ, β to low-γ, θ to high-γ, α to high-γ, or β to high-γ), sACS amplitude (‘amp’: 50, 100, 200, or 400 μA), and sACS frequency (‘freq’: 5, 10, 20, or 40 Hz).

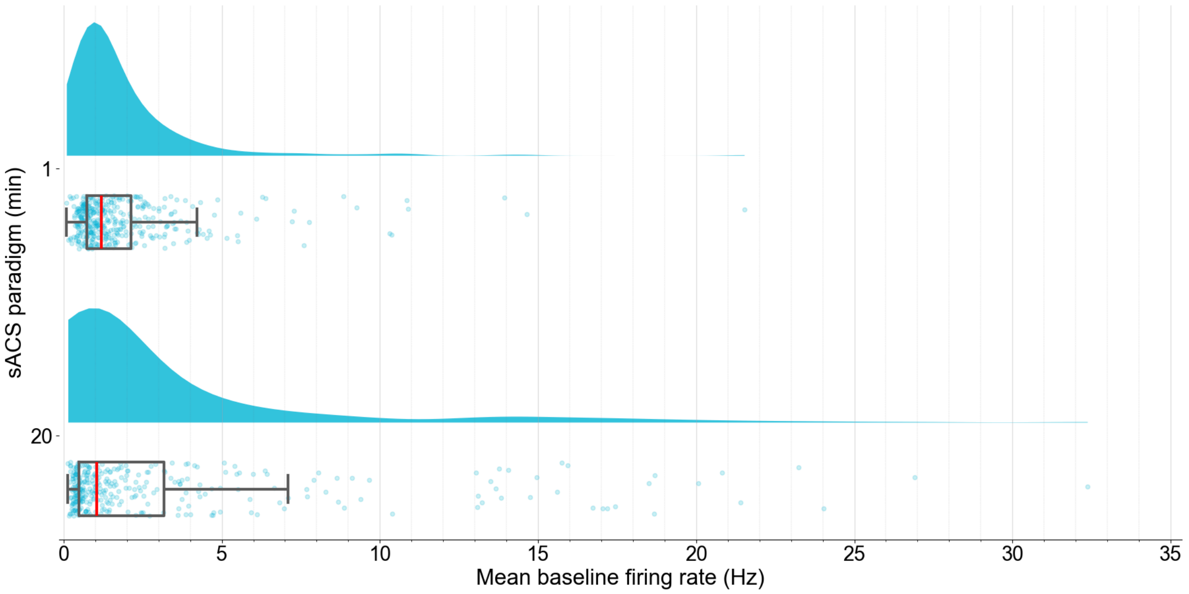

Figure S1.1. Baseline firing rates.

For 1- and 20-min sACS paradigms, raincloud plots showing the mean baseline firing rates for all neurons across all rats during baseline periods. Red bars indicate median values, with the boxplot showing the quartiles of the dataset. Neurons from 1-min experiments had a median firing rate of 1.20 ± 2.11 Hz, and those from 20-min experiments had a median firing rate of 1.05 ± 4.92 Hz.

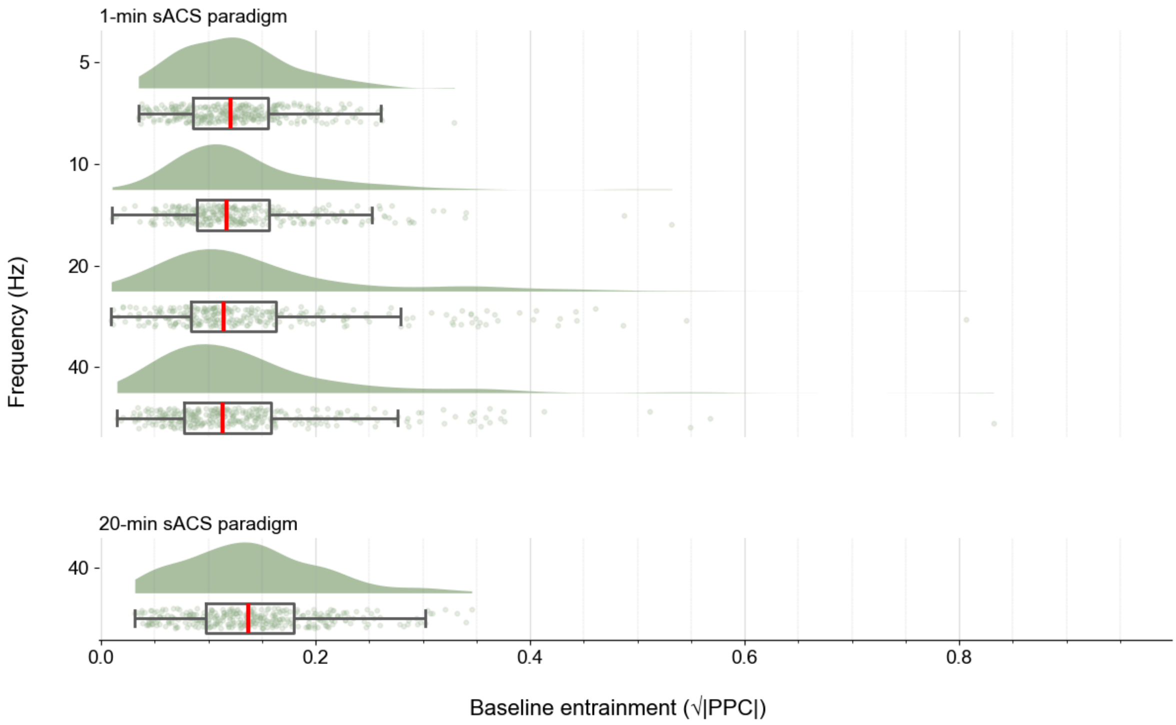

Figure S1.2. Baseline entrainment levels.
For 1- and 20-min sACS paradigms, raincloud plots showing the mean entrainment values (√|PPC|) for all neurons across all rats during baseline periods. Red bars indicate median values, with the boxplot showing the quartiles of the dataset. Neurons from 1-min experiments had median √|PPC| of 0.12 ± 0.05, 0.12 ± 0.07, 0.11 ± 0.10, 0.11 ± 0.09 to 5, 10, 20, and 40 Hz endogenous oscillatory frequencies, respectively, and those from 20-min experiments had a median √|PPC| of 0.14 ± 0.06 to 40 Hz frequencies.

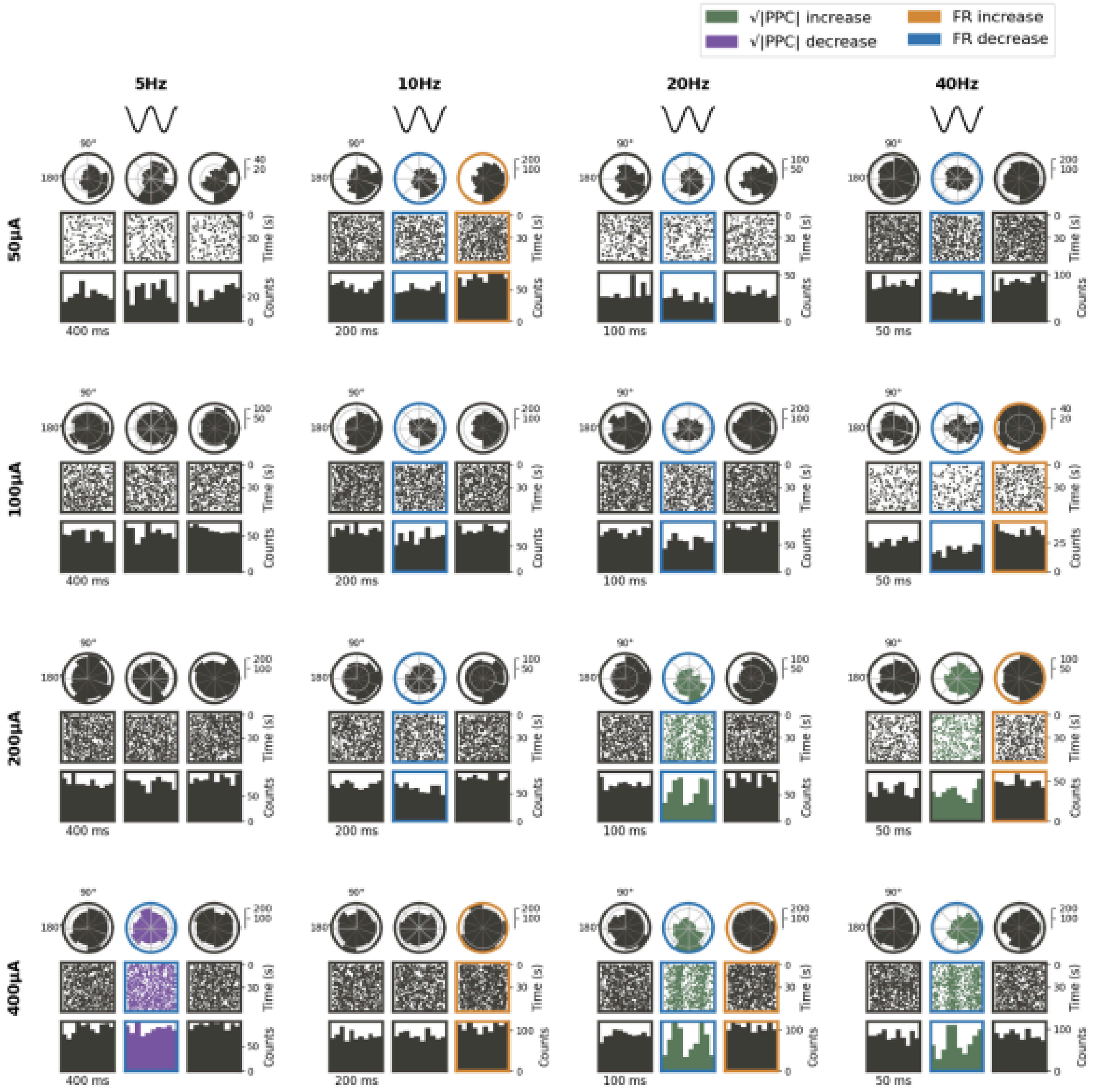

Figure S1.3. Exemplar neuron response across stimulation parameters, showing significantly increased entrainment at high frequency (20&40 Hz) and amplitude (100&200 µA) sACS.
A subplot is represented for each frequency (row) and amplitude (column). Blank subplots indicate the neuron did not meet the minimum ≥0.25 Hz FR during that condition. Within each subplot, the three columns show behaviour over one minute periods of pre-stimulus baseline, sACS stimulation period, and the period immediately succeeding sACS offset. The first row contains spike density histograms vs phase; the second brown, spike rasters; and the final row, spike density histograms vs time.

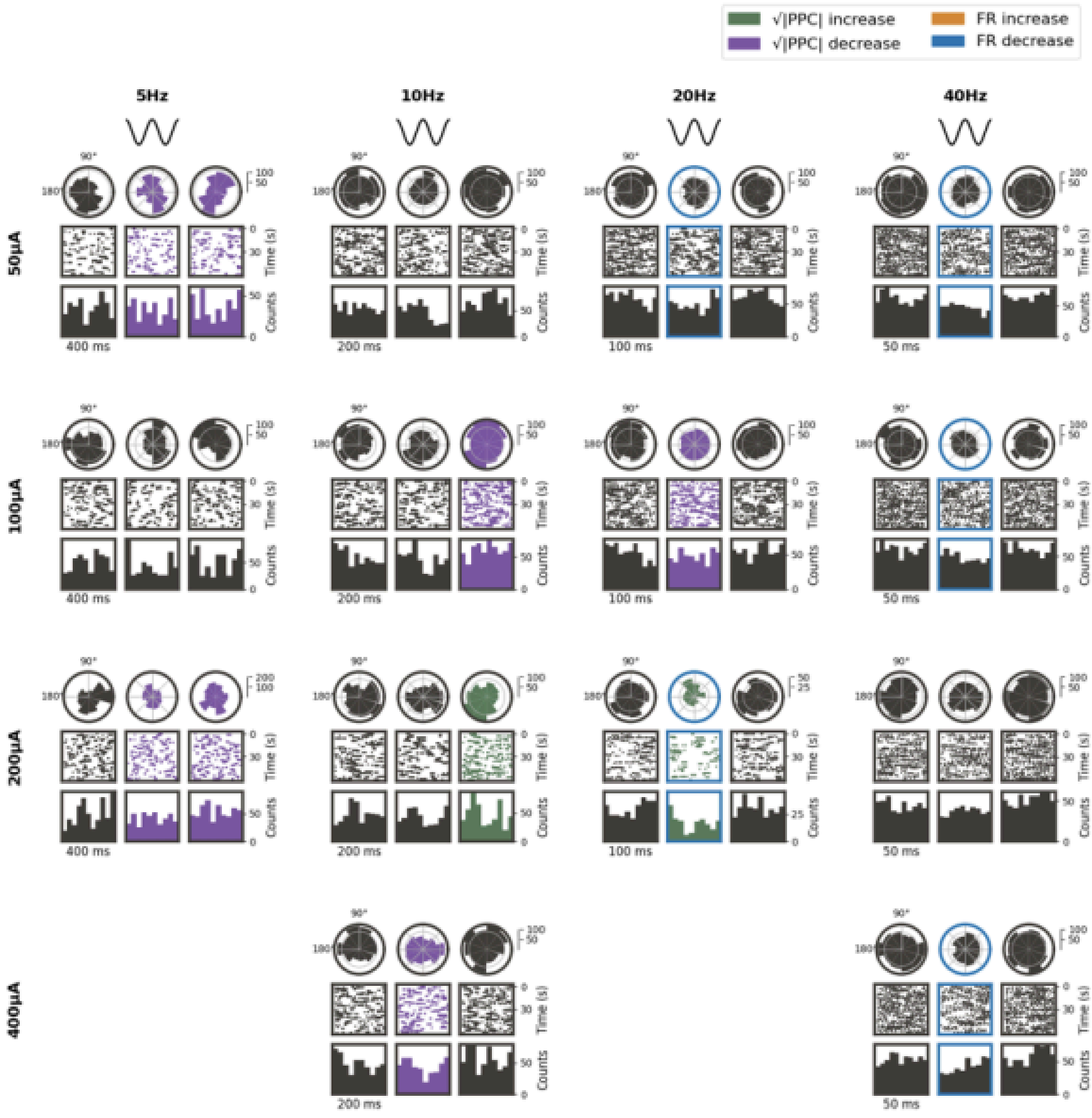

Figure S1.4. Exemplar neuron response across stimulation parameters, showing significantly decreased entrainment at various amplitudes and frequencies of sACS.

A subplot is represented for each frequency (row) and amplitude (column). Blank subplots indicate the neuron did not meet the minimum ≥0.25 Hz FR during that condition. Within each subplot, the three columns show behaviour over one minute periods of pre-stimulus baseline, sACS stimulation period, and the period immediately succeeding sACS offset. The first row contains spike density histograms vs phase; the second brown, spike rasters; and the final row, spike density histograms vs time.

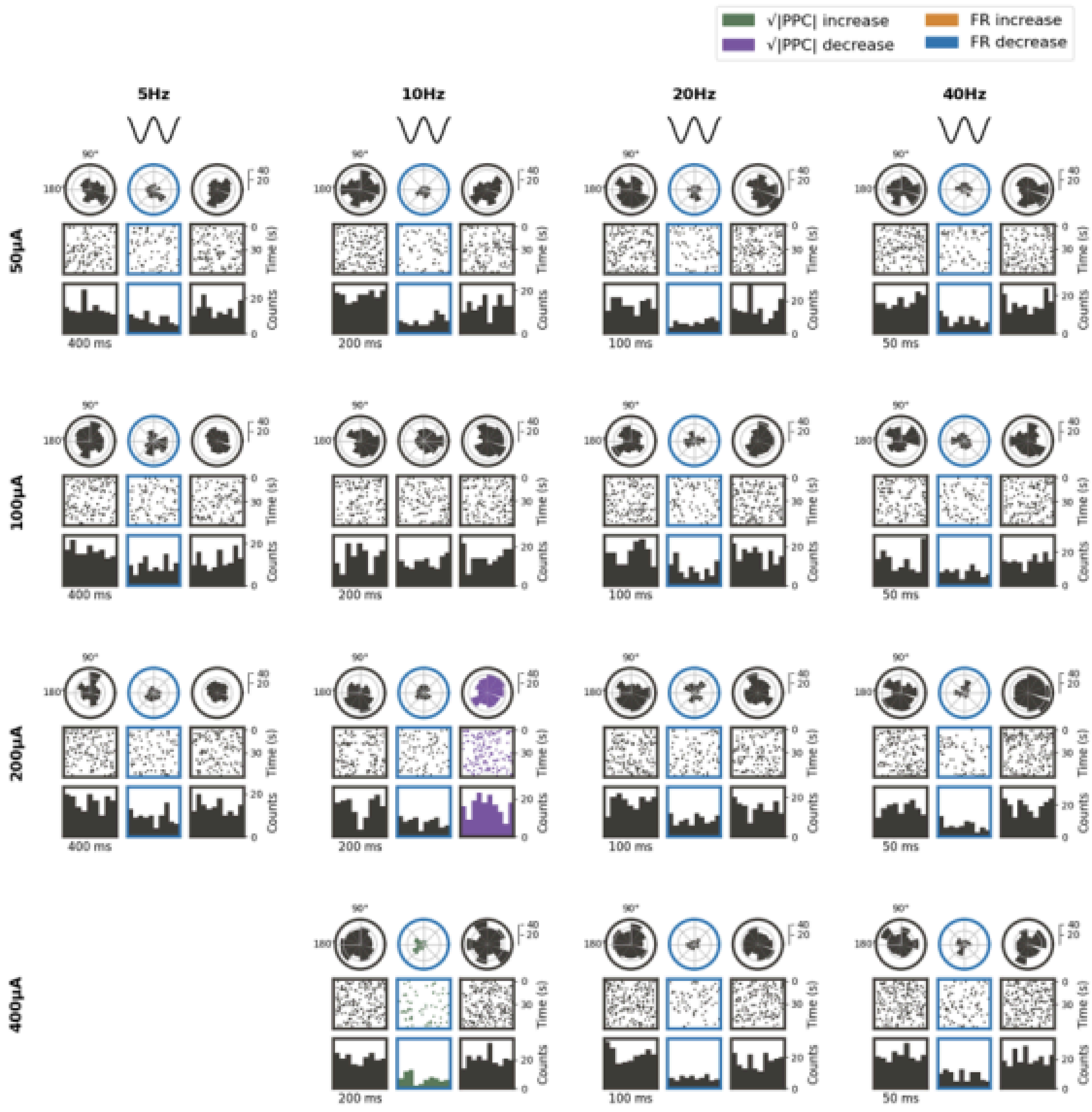

Figure S1.5. Exemplar neuron response across stimulation parameters, showing significantly decreased firing rates under sACS.
A subplot is represented for each frequency (row) and amplitude (column). Blank subplots indicate the neuron did not meet the minimum ≥0.25 Hz FR during that condition. Within each subplot, the three columns show behaviour over one minute periods of pre-stimulus baseline, sACS stimulation period, and the period immediately succeeding sACS offset. The first row contains spike density histograms vs phase; the second brown, spike rasters; and the final row, spike density histograms vs time.

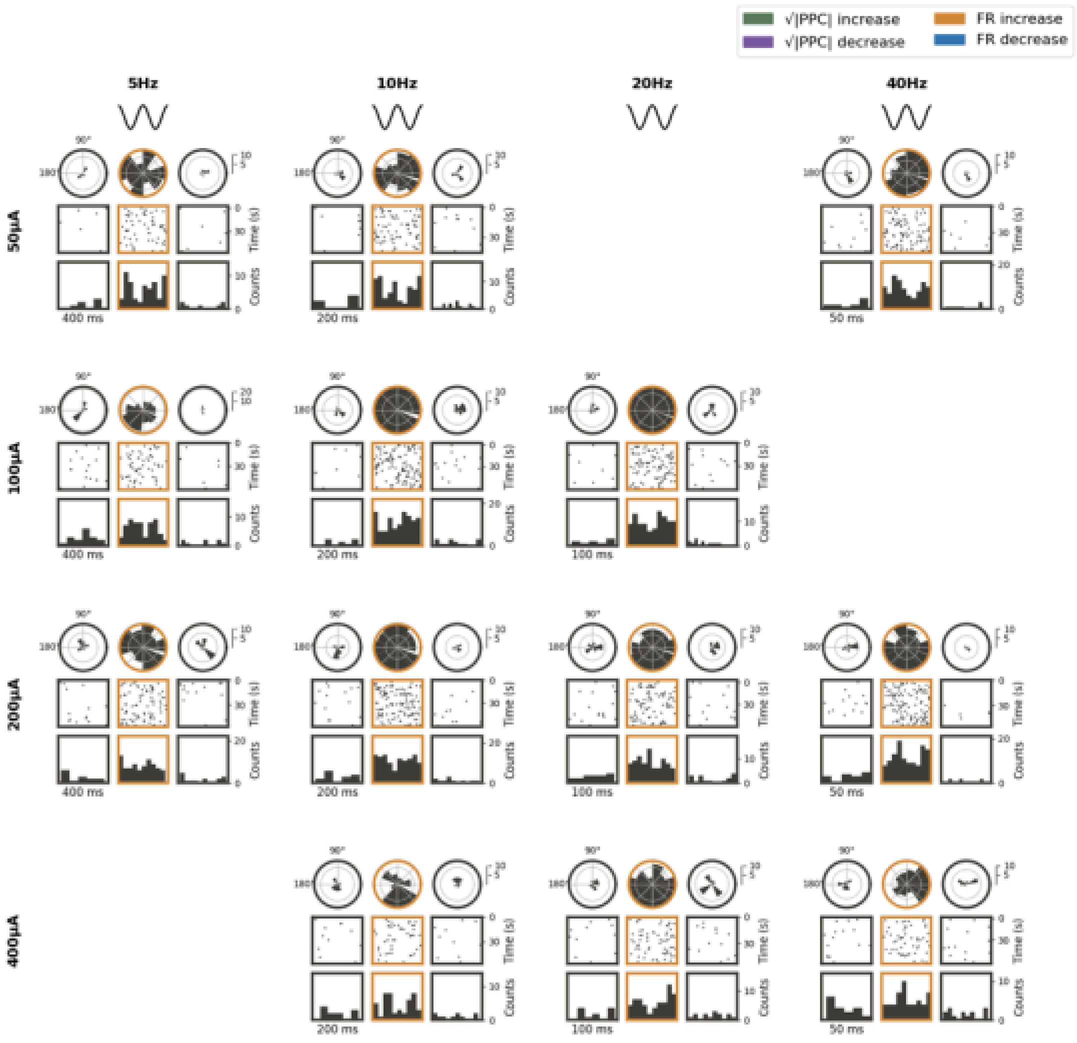

Figure S1.6. Exemplar neuron response across stimulation parameters, showing significantly increased firing rates under sACS.
A subplot is represented for each frequencies (row) and amplitudes (column). Blank subplots indicate the neuron did not meet the minimum ≥0.25 Hz FR during that condition. Within each subplot, the three columns show behaviour over one minute periods of pre-stimulus baseline, sACS stimulation period, and the period immediately succeeding sACS offset. The first row contains spike density histograms vs phase; the second brown, spike rasters; and the final row, spike density histograms vs time.

S2 *20-min sACS responses*

Spike timing metric	Frequency band	Log power pre sACS	Log power post sACS	eff_size	95% CI	emmean	SE of emmean	p	Signif
√|*PPC*| *increase*	δ	-14.71 ± 0.39	-15.12 ± 0.50	-5.45E-01	1.04E-01	-1.22E-01	1.12E-02	1.07E-24	***
	θ	-15.44 ± 0.37	-15.94 ± 0.59	-6.77E-01	1.04E-01	-1.51E-01	1.12E-02	3.27E-37	***
	α	-15.88 ± 0.36	-15.94 ± 0.52	-5.85E-01	1.04E-01	-1.31E-01	1.12E-02	3.59E-28	***
	β	-16.78 ± 0.31	-16.72 ± 0.35	-2.50E-01	1.04E-01	-5.58E-02	1.12E-02	2.63E-06	***
	low-γ	-18.23 ± 0.30	-18.17 ± 0.34	-1.97E-01	1.04E-01	-4.40E-02	1.12E-02	2.11E-04	***
	high-γ	-19.36 ± 0.17	-19.29 ± 0.28	-5.51E-02	1.04E-01	-1.23E-02	1.12E-02	3.00E-01	
√|*PPC*| *decrease*	δ	-14.79 ± 0.59	-15.08 ± 0.64	-5.26E-02	1.47E-01	-1.18E–02	1.68E-02	4.84E-01	
	θ	-15.82 ± 0.74	-15.99 ± 0.68	1.27E-01	1.47E-01	2.85E-02	1.68E-02	9.03E-02	
	α	-16.24 ± 0.70	-16.25 ± 0.63	2.43E-01	1.47E-01	5.43E-02	1.68E-02	1.23E-03	**
	β	-17.12 ± 0.60	-17.03 ± 0.54	3.93E-01	1.47E-01	8.77E-02	1.68E-02	1.77E-07	***
	low-γ	-18.70 ± 0.60	-18.46 ± 0.48	4.12E-01	1.47E-01	9.20E-02	1.68E-02	4.35E-08	***
	high-γ	-19.52 ± 0.45	-19.54 ± 0.36	2.55E-01	1.47E-01	5.70E-02	1.68E-02	6.98E-04	**
FR increase	δ	-15.18 ± 0.61	-15.20 ± 0.49	1.76E-01	4.09E-02	4.04E-02	4.78E-03	2.98E-17	***
	θ	-15.88 ± 0.60	-15.75 ± 0.50	3.31E-01	4.09E-02	7.59E-02	4.78E-03	9.67E-57	***
	α	-16.25 ± 0.59	-16.04 ± 0.54	4.30E-01	4.09E-02	9.86E-02	4.78E-03	1.65E-94	***
	β	-17.09 ± 0.50	-16.86 ± 0.45	4.50E-01	4.09E-02	1.02E-01	4.78E-03	2.77E-103	***
	low-γ	-18.50 ± 0.49	-18.28 ± 0.43	3.93E-01	4.09E-02	9.02E-02	4.78E-03	2.56E-79	***
	high-γ	-19.72 ± 0.36	-19.59 ± 0.34	2.34E-01	4.09E-02	5.36E-02	4.78E-03	3.27E-29	***
FR decrease	δ	-14.97 ± 0.52	-15.40 ± 0.49	-5.95E-01	5.26E-02	-1.36E-01	6.16E-03	9.70E-109	***
	θ	-15.72 ± 0.59	-16.33 ± 0.73	-8.46E-01	5.26E-02	-1.94E-01	6.16E-03	5.17E-218	***
	α	-16.02 ±	-16.52 ±	-7.83E-0	5.26E-02	-1.79E-0	6.16E-03	1.05E-18	***

		0.63	0.79	1		1		6	
	β	-16.85 ± 0.54	-17.18 ± 0.67	-6.77E-01	5.26E-02	-1.55E-01	6.16E-03	4.25E-140	***
	low-γ	-18.38 ± 0.48	-18.69 ± 0.51	-5.84E-01	5.26E-02	-1.34E-01	6.16E-03	7.15E-105	***
	high-γ	-19.52 ± 0.26	-19.82 ± 0.32	-4.03E-01	5.26E-02	-9.25E-02	6.16E-03	5.76E-51	***

Table S2.1 Pairwise comparisons of log power near spike timing affected neurons pre- and post- 20-min sACS.

Across all rats, the median log power and median absolute deviation pre- and post- 20-min sACS for electrode channels proximal to neurons with significant increases/decreases in entrainment/firing rate. Pairwise comparisons of the LMER were used to report the effect size and 95% confidence interval as well as the estimated marginal means, standard error, and p-value.

Spike timing metric	Phase frequency band	Amplitude frequency band	Order normalized MI pre sACS	Order normalized MI post sACS	eff_size	95% CI	emmean	SE of emmean	p	Signif
√|*PPC*| *increase*	δ	low-γ	1.26 ± 0.68	0.64 ± 0.78	-6.84E-01	2.31E-01	-3.93E-01	6.77E-02	6.40E-09	***
	θ	low-γ	0.50 ± 0.49	0.13 ± 0.62	-3.65E-01	2.31E-01	-2.10E-01	6.77E-02	1.95E-03	**
	α	low-γ	-0.24 ± 0.39	-0.24 ± 0.50	-3.71E-02	2.31E-01	-2.13E-02	6.77E-02	7.53E-01	
	β	low-γ	-1.12 ± 0.31	-1.01 ± 0.29	1.36E-01	2.31E-01	7.80E-02	6.77E-02	2.49E-01	
	δ	high-γ	1.10 ± 0.63	0.61 ± 0.86	-6.05E-01	2.31E-01	-3.47E-01	6.77E-02	2.87E-07	***
	θ	high-γ	0.65 ± 0.56	0.12 ± 0.78	-2.60E-01	2.31E-01	-1.50E-01	6.77E-02	2.71E-02	*
	α	high-γ	0.19 ± 0.43	0.17 ± 0.58	-5.16E-02	2.31E-01	-2.96E-02	6.77E-02	6.61E-01	
	β	high-γ	-0.65 ± 0.38	-0.60 ± 0.42	4.91E-02	2.31E-01	2.82E-02	6.77E-02	6.77E-01	
√|*PPC*| *decrease*	δ	low-γ	0.79 ± 0.66	0.71 ± 0.58	-2.87E-01	3.27E-01	-1.65E-01	9.57E-02	8.52E-02	
	θ	low-γ	0.22 ± 0.59	0.37 ± 0.46	2.34E-01	3.27E-01	1.34E-01	9.57E-02	1.61E-01	
	α	low-γ	-0.21 ± 0.69	-0.16 ± 0.58	1.39E-01	3.27E-01	8.01E-02	9.57E-02	4.02E-01	
	β	low-γ	-1.01 ± 0.42	-0.90 ± 0.39	1.11E-01	3.27E-01	6.37E-02	9.57E-02	5.06E-01	
	δ	high-γ	0.36 ± 0.67	0.50 ± 0.47	5.63E-02	3.27E-01	3.23E-02	9.57E-02	7.36E-01	
	θ	high-γ	0.10 ± 0.55	0.17 ± 0.61	1.30E-01	3.27E-01	7.44E-02	9.57E-02	4.37E-01	
	α	high-γ	-0.11 ± 0.72	0.07 ± 0.66	3.76E-01	3.27E-01	2.16E-02	9.57E-02	2.40E-02	*
	β	high-γ	-0.57 ± 0.53	-0.44 ± 0.54	2.20E-01	3.27E-01	1.26E-02	9.57E-02	1.87E-01	
FR increase	δ	low-γ	0.89 ± 0.71	1.00 ± 0.66	4.14E-02	9.06E-02	2.61E-02	2.91E-02	3.70E-01	
	θ	low-γ	0.51 ± 0.53	0.58 ± 0.49	4.20E-02	9.06E-02	2.65E-02	2.91E-02	3.64E-01	
	α	low-γ	-0.06 ± 0.55	0.09 ± 0.55	1.58E-01	9.06E-02	9.93E-02	2.91E-02	6.52E-04	**
	β	low-γ	-0.97 ± 0.47	-0.82 ± 0.47	1.61E-01	9.06E-02	1.02E-02	2.91E-02	4.89E-04	***
	δ	high-γ	0.59 ± 0.68	0.76 ± 0.56	2.30E-01	9.06E-02	1.45E-01	2.91E-02	6.29E-07	***

	**θ**	**high-γ**	0.48 ± 0.60	0.52 ± 0.55	7.12E-02	9.06E-02	4.49E-02	2.91E-02	1.23E-01	
	**α**	**high-γ**	0.14 ± 0.64	0.32 ± 0.63	1.96E-01	9.06E-02	1.24E-01	2.91E-02	2.20E-05	***
	**β**	**high-γ**	-0.51 ± 0.53	-0.35 ± 0.56	1.45E-01	9.06E-02	9.12E-02	2.91E-02	1.74E-03	**
FR decrease	**δ**	**low-γ**	0.55 ± 0.68	0.17 ± 0.44	-9.51E-01	1.17E-01	-5.99E-01	3.75E-02	2.12E-57	***
	**θ**	**low-γ**	0.22 ± 0.56	-0.17 ± 0.36	-5.88E-01	1.17E-01	-3.70E-01	3.75E-02	5.80E-23	***
	**α**	**low-γ**	-0.27 ± 0.41	-0.58 ± 0.42	-3.38E-01	1.17E-01	-2.13E-01	3.75E-02	1.42E-08	***
	**β**	**low-γ**	-1.11 ± 0.32	-1.12 ± 0.25	-9.83E-03	1.17E-01	-6.19E-04	3.75E-02	8.69E-01	
	**δ**	**high-γ**	0.32 ± 0.61	0.00 ± 0.52	-8.57E-01	1.17E-01	-5.40E-01	3.75E-02	5.51E-47	***
	**θ**	**high-γ**	0.16 ± 0.53	-0.18 ± 0.34	-5.72E-01	1.17E-01	-3.60E-01	3.75E-02	7.96E-22	***
	**α**	**high-γ**	-0.10 ± 0.46	-0.44 ± 0.47	-4.64E-01	1.17E-01	-2.93E-01	3.75E-02	6.43E-15	***
	**β**	**high-γ**	-0.70 ± 0.33	-0.78 ± 0.33	-1.52E-01	1.17E-01	-0.60E-02	3.75E-02	1.05E-02	***

Table S2.2. Pairwise comparison of order normalized modulation indices near spike timing affected neurons pre- and post- 20-min sACS.

Across all rats, the median order normalized modulation index and median absolute deviation pre- and post- 20-min sACS for electrode channels proximal to neurons with significant increases/decreases in entrainment/firing rate. Pairwise comparisons of the LMER were used to report the effect size and 95% confidence interval as well as the estimated marginal means, standard error, and p-value.

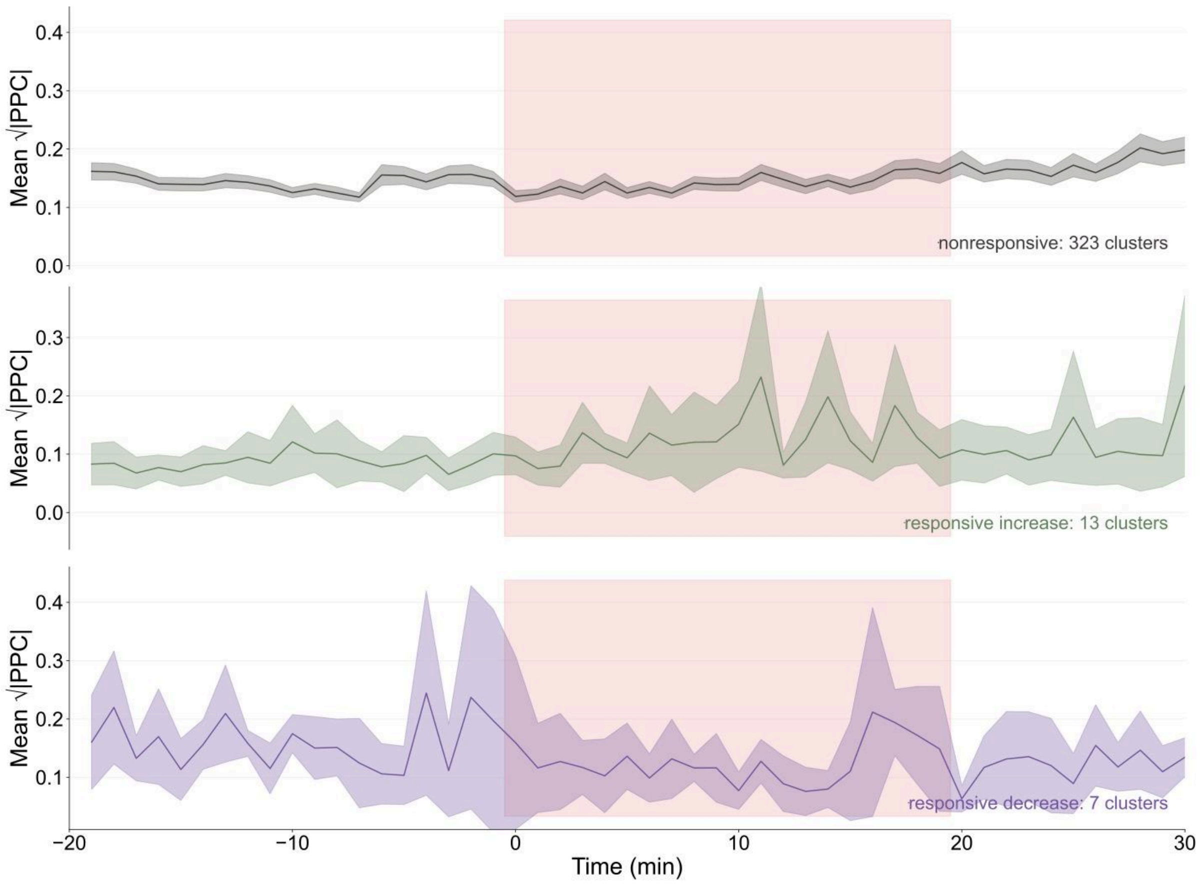

Figure S2.1. Entrainment over time for neurons significantly affected when comparing the full 20 minutes of stimulation against 20 minutes of preceding baseline.

√|PPC|s were computed over one minute windows and plotted over time for neurons: without significant √|PPC| changes (grey, n = 323), increased √|PPC| (green, n =13), and decreased √|PPC| (purple, n = 7). Shaded area indicates 95% CI.

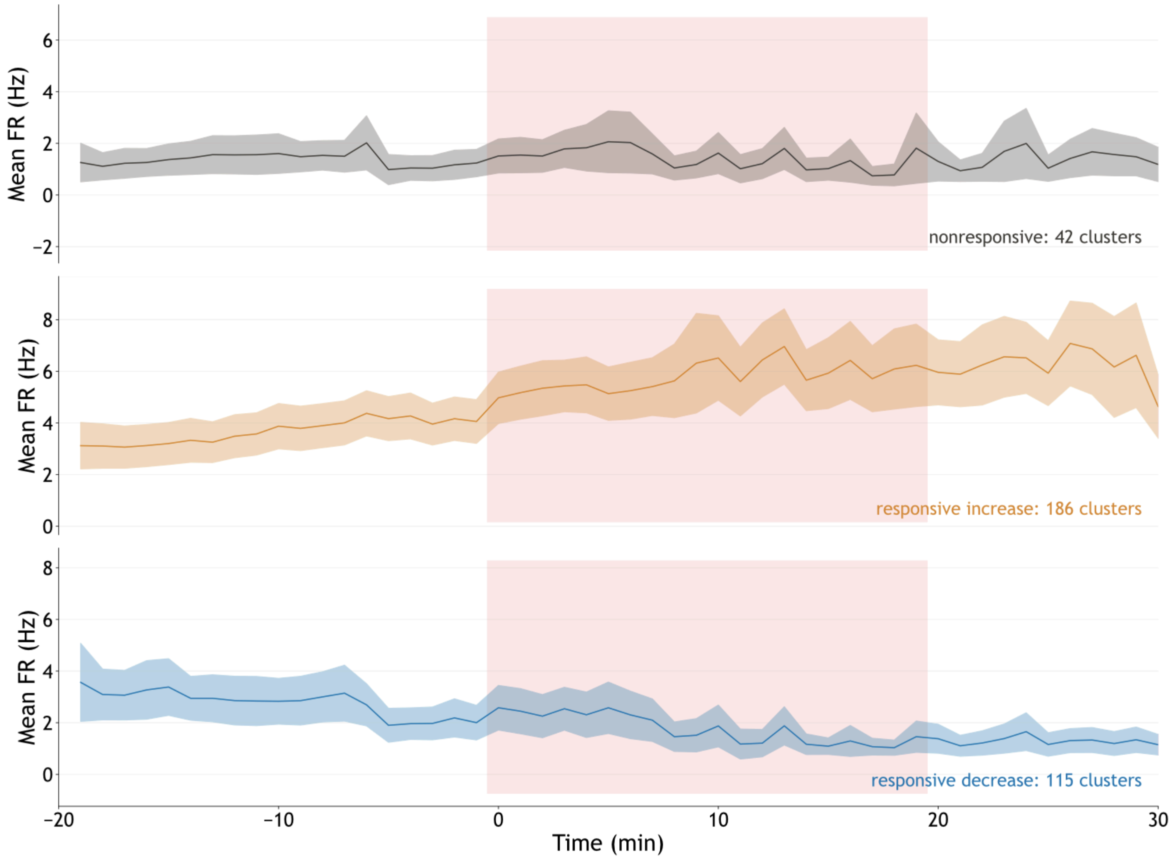

Figure S2.2. Firing rates over time for neurons significantly affected when comparing the entire 20 minutes of stimulation against 20 minutes of preceding baseline.

FRs were computed over one minute windows and plotted over time for neurons: without significant FR changes (grey, n = 42), increased FR (orange, n = 186), and decreased FR (blue, n = 115). Shaded area indicates 95% CI.

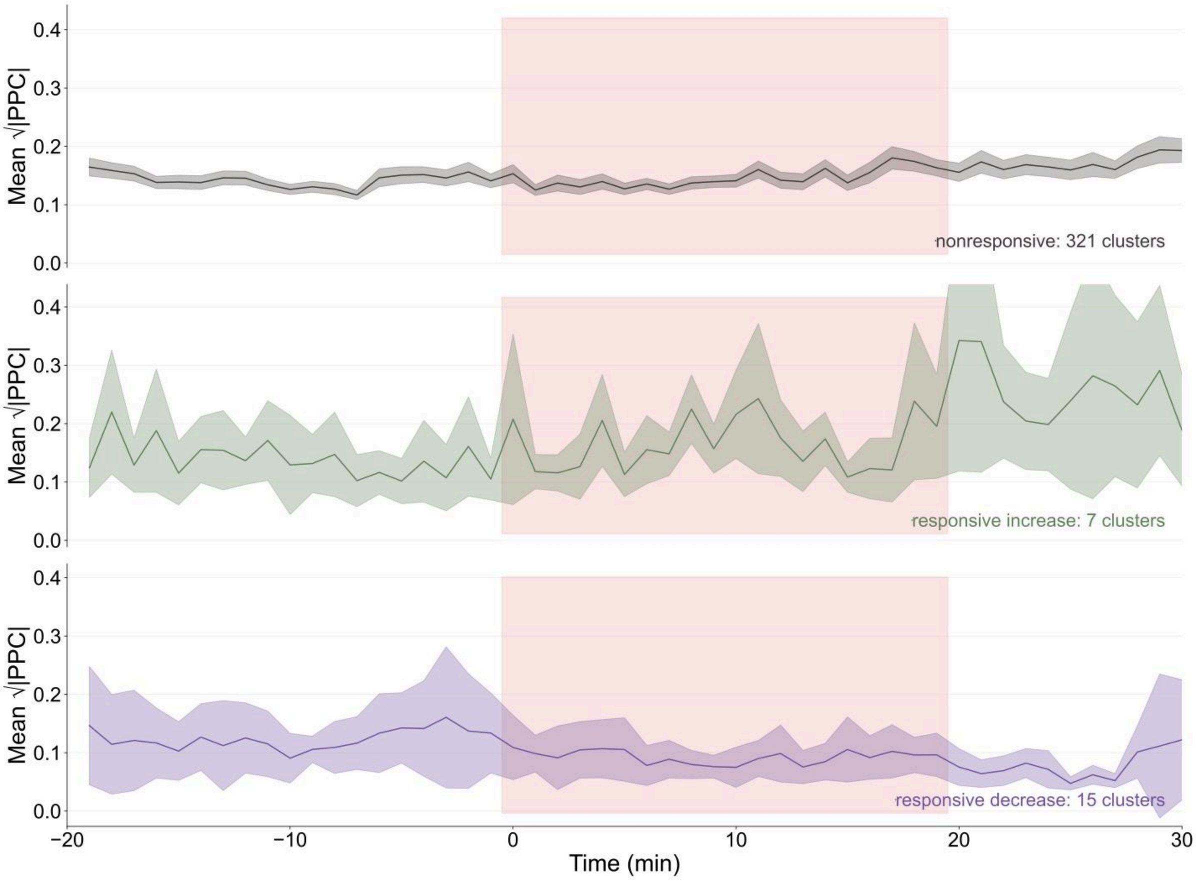

Figure S2.3. Entrainment over time for neurons significantly affected when comparing 10 minutes immediately post stimulation against 10 minutes immediately preceding stimulation.

√|PPC|s were computed over one minute windows and plotted over time for neurons: without significant √|PPC| changes (grey, n = 321), increased √|PPC| (green, n = 7), and decreased √|PPC| (purple, n = 15). Shaded area indicates 95% CI.

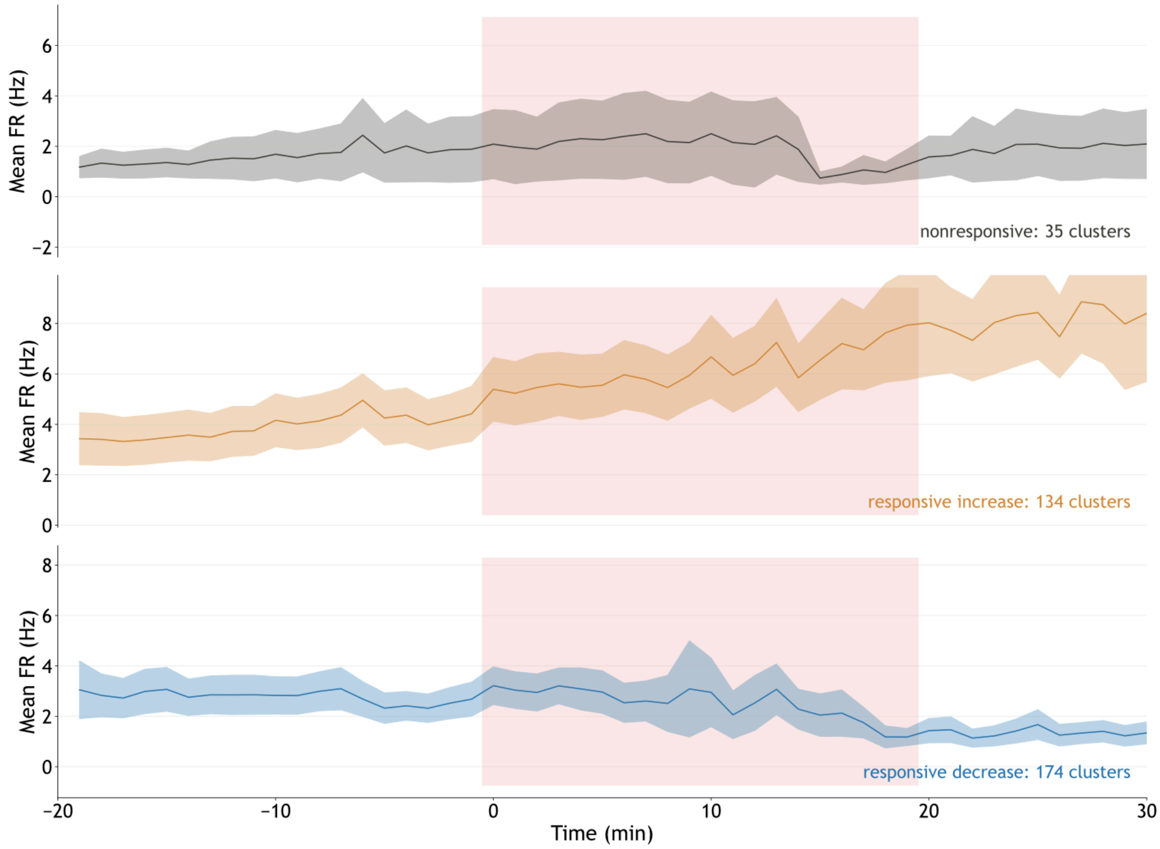

Figure S2.4. Firing rates over time for neurons significantly affected when comparing 10 minutes immediately post stimulation against 10 minutes immediately preceding stimulation.

FRs were computed over one minute windows and plotted over time for neurons: without significant FR changes (grey, n = 35), increased FR (orange, n = 134), and decreased FR (blue, n = 174). Shaded area indicates 95% CI.

S3 ***Sham sACS responses***

			Amplitude (μA)			
			**50**	**100**	**200**	**400**
**Frequency (Hz)**	**5**	*√|PPC| increase*	0/54	0/19	2/53	2/30
		√|PPC| decrease	1/54	1/19	1/53	1/30
		FR increase	0/54	0/19	0/53	0/30
		FR decrease	0/54	0/19	0/53	0/30
	**10**	*√|PPC| increase*	0/44	0/31	0/15	0/31
		√|PPC| decrease	0/44	1/31	0/15	1/31
		FR increase	0/44	0/31	0/15	0/31
		FR decrease	0/44	0/31	0/15	0/31
	**20**	*√|PPC| increase*	2/27	2/42	0/31	2/47
		√|PPC| decrease	1/27	0/42	0/31	0/47
		FR increase	0/27	0/42	0/31	0/47
		FR decrease	0/27	0/42	0/31	0/47
	**40**	*√|PPC| increase*	0/64	0/36	1/36	0/32
		√|PPC| decrease	1/64	1/36	0/36	0/32
		FR increase	0/64	0/36	0/36	0/32
		FR decrease	0/64	0/36	0/36	0/32

Table S3.1. Number of neurons affected across sham 1-min stimulation paradigms.
For each amplitude and frequency combination of sham sACS delivered in a single rat, the number of neurons with firing of at rates of >= 0.25 Hz for that trial which were detected as being significantly modulated from the 1-min of preceding baseline.
